# Addressing the Need for a Preexposure Prophylaxis Monitoring and Evaluation Implementation Guide: Experience From Zambia

**DOI:** 10.9745/GHSP-D-22-00396

**Published:** 2023-04-28

**Authors:** Marie-Claude C. Lavoie, Natalie Blanco, Linah K. Mwango, Brooke E. Nichols, Anna Whittington, Brianna Lindsay, Olufunso Adebayo, Morley Mujansi, Kalima Tembo, Lottie Hachaambwa, Daliso Mumba, Bupe Musonda, Cassidy W. Claassen

**Affiliations:** a Division of Global Health Sciences, Department of Epidemiology and Public Health, University of Maryland School of Medicine, Baltimore, MD, USA.; b Institute of Human Virology, University of Maryland School of Medicine, Baltimore, MD, USA.; c Center for International Health Education and Biosecurity, University of Maryland School of Medicine, Baltimore, MD, USA.; d Center for International Health Education and Biosecurity-Zambia, Lusaka, Zambia.; e Department of Global Health, School of Public Health, Boston University, Boston, Massachusetts, USA.; f Maryland Global Initiatives Corporation Zambia, Lusaka, Zambia.; g National HIV/AIDS/STI/TB Council, Lusaka, Zambia.; h Zambia Ministry of Health, Lusaka, Zambia.

## Abstract

**Background:**

The HIV care continuum comprises well-defined steps and indicators. In contrast, indicators along the preexposure prophylaxis (PrEP) cascade are still in the early stages of implementation. Monitoring and evaluation (M&E) of PrEP services is critical to optimizing PrEP uptake and adherence during periods of HIV risk. We provide an overview of global indicators for PrEP, describe the development process and outcomes of Zambia’s 2022 *National Pre-Exposure Prophylaxis (PrEP) Program Monitoring & Evaluation Implementation Guide*, and discuss the guide’s implications for other countries in sub-Saharan Africa.

**National M&E Guide Development Process:**

During the scale-up of PrEP, the Zambia Ministry of Health (MOH) and the National HIV/AIDS/STI/TB Council recognized the need for a national unified monitoring system to guide the effective implementation of PrEP services. Stakeholders from the MOH, civil society, professional organizations, funding agencies, and implementing partners developed the *National Pre-Exposure Prophylaxis (PrEP) Program Monitoring & Evaluation Implementation Guide*. This guide is aligned with the existing global indicators from the World Health Organization and the U.S. President’s Emergency Plan for AIDS Relief and adapted to the country’s needs, context, and health information systems. Zambia’s experience in developing the guide has highlighted the importance of strengthening client-level monitoring systems for HIV prevention, ensuring flexibility of the PrEP monitoring system to accommodate PrEP delivery modalities and differentiated service delivery models, and training health workers to enhance PrEP services and deliver care PrEP services along the continuum to prevent HIV acquisition effectively.

**Conclusions:**

Using a collaborative and consensus-based approach, Zambia developed its first national PrEP M&E implementation guide to provide standardized guidelines for optimizing the delivery, monitoring, and evaluation of PrEP service delivery. Zambia’s experience can inform other countries in sub-Saharan Africa as they develop national M&E implementation approaches for PrEP.

## Abstract (precis)

The authors share the experience of developing a national preexposure prophylaxis program monitoring and evaluation implementation guide in Zambia, which can inform approaches to meet similar needs in other sub-Saharan African countries.

## Teaser

**Key Findings:**

- Under the leadership of the Zambia Ministry of Health, stakeholders developed a national monitoring and evaluation (M&E) implementation guide to measure the uptake, use, safety, and cost of preexposure prophylaxis (PrEP).
- The guide builds upon global indicators and incorporates specific indicators to meet the country’s needs and provide a comprehensive overview of the PrEP cascade. The development and use of indicators along the PrEP care continuum are still in the initial stages in most countries.

**Key Implication:**

- As other countries scale up PrEP services and plan for a robust M&E program, they should consider the following: implementation of a patient-level health information system, use of a people-centered approach that allows flexibility for PrEP modalities and location of service delivery (both community and facility), and training of health care workers.

## BACKGROUND

From 2020 to 2021, the number of new HIV infections decreased globally by 31%.^1^ However, in 2020 alone, 1.5 million people acquired HIV, far short of the global target of less than 500,000 new infections per year.^1^ The World Health Organization (WHO) recommends that preexposure prophylaxis (PrEP) be part of a combination HIV prevention program for people at risk of HIV infection.^2^ This recommendation aligns with the mounting evidence demonstrating the efficacy and safety of PrEP among different populations.^3^^–^^6^ Daily oral PrEP has been shown to decrease the risk of HIV acquisition by 90% or more.^3^^,^^7^^,^^8^ PrEP use should be aligned with periods of increased HIV risk; in contrast, antiretroviral therapy requires lifelong adherence to prevent HIV-related morbidity, mortality, and transmission. The term “prevention‐effective adherence” is commonly used to describe the importance of taking PrEP as indicated during periods of HIV risk, which may fluctuate over time.^9^ HIV incidence is higher among individuals discontinuing or experiencing gaps in PrEP use than individuals on PrEP continuously.^10^

The HIV care continuum comprises defined steps—from HIV diagnosis to antiretroviral therapy initiation to viral suppression—accompanied by well-defined indicators.^11^ In contrast, the development and use of indicators along the PrEP care continuum are still in the initial stages in most countries. Routine monitoring and robust evaluations are critical to examining and optimizing the access, availability, uptake, adherence, and effectiveness of PrEP in real-world settings.^12^ Robust PrEP program data can also be used to inform the procurement and financing of health care commodities to prevent stock-outs and disruption of service delivery.

Routine monitoring and robust evaluations are critical to examine and ensure the access, availability, uptake, adherence, and effectiveness of PrEP in real-world settings.

In 2017, during the early phase of PrEP rollout in sub-Saharan Africa (SSA), Zambia was one of the first countries to include PrEP as part of its combination HIV prevention efforts to curb the number of new HIV infections, estimated at 75,000.^13^ As the country scaled up PrEP, the Ministry of Health (MOH) recognized the need for a comprehensive and unified PrEP monitoring and evaluation (M&E) guide to measure and monitor access, availability, and PrEP continuation (i.e., PrEP refill) and to ensure adequate financing to achieve optimal impact. In this commentary, we provide an overview of global indicators for PrEP, describe the process and outcomes of Zambia’s 2022 *National Pre-Exposure Prophylaxis (PrEP) Program Monitoring & Evaluation Implementation Guide* (hereafter referred to as the National PrEP Program M&E Implementation Guide), and discuss its implications for other countries in SSA.^14^ The guide is included as a Supplement.

## NATIONAL M&E GUIDE DEVELOPMENT PROCESS

### Overview of PrEP Indicators

Since 2003, the U.S. President’s Emergency Plan for AIDS Relief (PEPFAR) has been instrumental in the HIV global response. PEPFAR supports more than 50 countries, including Zambia, to achieve HIV epidemic control. To maintain a coordinated and data-driven HIV response, PEPFAR has invested in and supported national health information systems to collect HIV-related data. In 2017, PEPFAR introduced a cross-program PrEP indicator that measured the number of individuals newly initiated on PrEP, known as PrEP_NEW.^15^ In 2018, PEPFAR added a second PrEP indicator to include the total number of individuals who were currently receiving PrEP, referred to as PrEP_CURR, which was later replaced by the number of individuals returning for follow-up or reinitiation visits (PrEP_CT).^16^^,^^17^ These indicators (PrEP_NEW and PrEP_CT) provide an ecological overview of PrEP uptake and rollout. WHO has also proposed PrEP indicators as part of its 2017 *Implementation Tool for Pre-exposure Prophylaxis (PrEP) of HIV Infection.*^18^ These indicators measure salient aspects of PrEP program cascades, such as PrEP early continuation, toxicity prevalence, and HIV seroconversion among PrEP users.^18^ Like PEPFAR, WHO suggests collecting these indicators disaggregated by sex, age group, and type of key population (e.g., female sex workers, men who have sex with men, or persons who inject drugs). Program implementers, MOH staff, and donors use this level of disaggregation to examine the uptake and reach of PrEP across subpopulations and implement data-driven and tailored interventions.

### Zambia National PrEP Program M&E Implementation Guide

According to the latest 2021 UNAIDS statistics, more than 147,397 individuals in Zambia were receiving PrEP services, representing a major increase from 3,823 individuals in 2018.^19^ A major increase in PrEP recipients in Zambia between 2018 and 2020 prompted the need for a unified monitoring system across the entire PrEP cascade. To address this implementation gap, the MOH and the National HIV/AIDS/STI/TB Council convened a series of stakeholder meetings in 2019–2020 with civil society groups, professional organizations, and funding agencies—including the U.S. Agency for International Development; U.S. Centers for Disease Control and Prevention; WHO; Global Fund to Fight AIDS, Tuberculosis and Malaria; and PEPFAR implementing partners, which include the Center for International Health, Education, and Biosecurity at the University of Maryland Baltimore (Ciheb-UMB); Ciheb-Zambia; Centre for Infectious Disease Research Zambia; John Snow International; and others. The primary objectives of these consultations were to develop a national PrEP M&E implementation guide to measure the uptake, use, safety, and cost of PrEP and to assess how to integrate it with the existing national health information system and data collection tools. These consultation meetings were held virtually due to the COVID-19 pandemic and hosted by Ciheb-UMB, on a virtual platform, monthly at first and then quarterly thereafter. After the 2021 national election, additional consultations were held with the government to refine the guide. In 2022, the Global Fund hosted an in-person meeting with all stakeholders to finalize and approve the document. The MOH, funding agencies, and PEPFAR implementing partners unanimously approved it in 2022.

A major increase in PrEP recipients in Zambia between 2018 and 2020 prompted the need for a unified monitoring system across the entire PrEP cascade.

**Figure:**
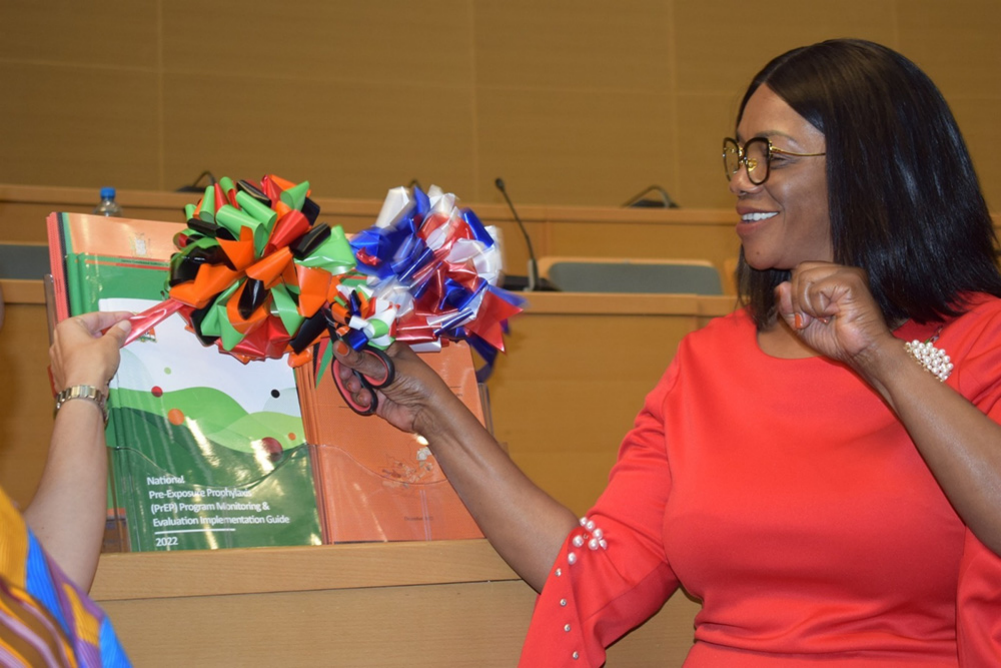
The Honorable Sylvia Masebo, Minister of Health and Member of Parliament, launches the *National Pre-Exposure Prophylaxis (PrEP) Program Monitoring & Evaluation Implementation Guide* during the 2022 World AIDS Day commemoration in Lusaka, Zambia. © 2022 Man Chilu Sounds Entertainment & Creative Arts

The National PrEP Program M&E Implementation Guide aligns with the existing global indicators from the WHO and PEPFAR.^17^^,^^20^ The guide includes indicators to measure the number of individuals screened for PrEP services, number of individuals eligible for PrEP (Box), number of individuals initiated on PrEP and who continued PrEP beyond 3 months, reasons for discontinuation, and cost-effectiveness of the program. These data can be used to identify populations, facilities, and geographic locations where PrEP demand-creation activities or PrEP services need to be optimized. The guide includes indicators that can be prioritized based on the needs, infrastructure, and human and financial resources available to collect, analyze, and interpret the data. In the Table, we outline M&E questions that can be answered by the indicators provided in the PEPFAR MER Guidance, WHO PrEP Implementation Tool, and Zambia National PrEP Program M&E Implementation Guide.

**TABLE:** Sample PrEP Evaluation Questions by Availability of Answers in Selected M&E Resources

Evaluation Questions	PEPFAR MER	WHO PrEP M&E	**Zambia PrEP M&EImplementation Guide**
Is PrEP reaching the intended population groups?^a^	X	X	X
Are subpopulations that might benefit from PrEP underrepresented among those receiving PrEP?^a^	X	X	X
Are people who are offered PrEP choosing to take it?^a^		X	X
Was PrEP effective in keeping clients HIV-negative?^a^		X	X
What are the forecasted PrEP-associated costs for the coming year?		X	X
What is the proportion of individuals alternating in/out of PrEP?			X
What is the proportion of PrEP users who continue using PrEP for more than 3 consecutive months and beyond?			X
What are patterns of PrEP use?^a^			X
How cost-effective is the program, and are there opportunities to improve efficiency?^a^			X
Among HIV negative clients, what is the proportion of individuals who are screened and eligible for PrEP?			X
Has HIV incidence decreased in the community?^a^		X	X

Abbreviations: M&E, monitoring and evaluation; MER, monitoring, evaluation, and reporting; PEPFAR, U.S. President's Emergency Plan for AIDS Relief; PrEP, preexposure prophylaxis; WHO, World Health Organization.

^a^Evaluation question examples proposed in the WHO PrEP monitoring framework.^21^

BOX Zambia Preexposure Prophylaxis Risk-Based Eligibility Criteria (2022)

Persons at substantial risk for HIV infection, defined as engaging in one or more of the following activities within the last 6 months, are recommended for preexposure prophylaxis:

- Vaginal and/or anal intercourse without condoms with multiple sexual partners
- Sexually active with a partner who is known to be HIV-positive or at substantial risk of being HIV-positive
- Sexually active with an HIV-positive partner who is not on effective treatment (defined as on combination antiretroviral therapy for less than 6 months) or not virally suppressed (viral load higher than 1,000 copies/ml)
- History of sexually transmitted infections
- History of postexposure prophylaxis use
- Sharing injection material or equipment

### Implementation Considerations

Zambia’s experience in developing a national PrEP M&E implementation guide can assist other countries in establishing a similar national implementation guide aligned with global indicators and responsive to the local context. This experience is especially relevant because, as of 2020, the number of users initiating PrEP in SSA is 290,891, comprising 44% of global PrEP users.^22^ We present practical considerations that were part of the development process of the National PrEP Program M&E Implementation Guide.

#### Implement an Integrated Longitudinal Client-Level Monitoring System

Electronic health record (EHR) systems are increasingly used to deliver and manage clinical care, including HIV services, in resource-constrained settings.^23^ Health information systems can be used for the planning, delivery, and evaluation of health services and resource allocation.^24^^,^^25^ The use of EHRs has improved the efficiency of service delivery and data quality, reducing gaps along the HIV care continuum and evaluating programs to guide continuous improvement and decision-making.^26^^–^^29^ Adapting EHR systems and data collection tools to collect PrEP data along the PrEP cascade can improve data use and monitoring to optimize PrEP continuation and re-initiation when needed. EHRs may also assist in managing the laboratory monitoring required while on PrEP, including HIV testing, hepatitis B testing, monitoring of serum creatinine, and screening for sexually transmitted infections and acute HIV infection.^2^

In Zambia, the national EHR SmartCare has been primarily designed and funded to support HIV care by optimizing the efficiency and quality of care. SmartCare had been deployed to over 80% of government health facilities as of 2022. In Zambia, SmartCare has been expanded to include a PrEP module, addressing a common shortcoming in SSA, which is that EHR systems often lack data on non-HIV conditions or medications such as PrEP.^30^ SmartCare captures data on individuals along the PrEP care cascade from screening and eligibility to initiation, continuation, and discontinuation.^31^ SmartCare deployment continues to expand across facilities, while paper-based registers for aggregate data collection are still widely used. With the development of the new implementation guide, revisions and improvements were made to data collection registers and reflected in electronic systems to fully capture each step in the PrEP cascade (screening, eligibility, initiation, and PrEP refills) and monitor clients longitudinally to guide service delivery and improve health outcomes. It was crucial that data elements could be linked to their corresponding indicators. At the community level, PEPFAR implementing partners, such as Ciheb-UMB, have also developed a DHIS2 Tracker system^32^ to complement SmartCare data for PrEP delivery. Community health workers enter client-level data into DHIS2 Tracker using approved MOH forms. In both SmartCare and DHIS2 Tracker, the use of auto-generated unique identifiers and issuance of a National Unique Patient Identification Number both protect the client’s confidentiality and establish consistent tracking over time. Client-level longitudinal monitoring systems can assist in assessing factors associated with specific health outcomes (e.g., PrEP discontinuation) to guide program implementation promptly and identify specific groups at risk for PrEP discontinuation while at risk for HIV. Understanding the resources used (in terms of medication dispensed, interactions with health care providers, and diagnostic tests performed) is essential for PrEP program support activities, such as resource planning, supply chain management, and costing and financial planning, as well as for future cost-effectiveness analyses of different PrEP implementation modalities.^33^

In Zambia, a national PrEP task force reviews the latest clinical evidence related to PrEP, including PrEP medications, and ensures that health systems adopt these changes in terms of health information systems, guidelines, and training of health care workers. This organizational structure is well positioned to plan and coordinate PrEP services systematically as they evolve.

#### Adapt M&E Systems as PrEP Modality and Services Evolve

As of 2022, the medications licensed and approved for HIV PrEP in Zambia include the daily oral combination pills of TDF/FTC and TDF/3TC. However, as trials continue for additional types and formulations of PrEP—including long-lasting injectable forms of PrEP (e.g., cabotegravir)—more PrEP medications are likely to be commonly available in SSA.^34^ M&E systems for PrEP need to accommodate new types of PrEP modalities, which will impact the frequency and type of clinical services received. Independent of the PrEP modalities used, clients will require counseling and monitoring to optimize the continuous use of PrEP during the period of risk. Also, under a differentiated service model and people-centered approach—which promotes adapting services delivery to the client’s needs and preferences while alleviating the burden on the health system—PrEP services are likely to become more widely available in settings beyond health care facilities, such as community-based mobile clinics, community safe places, and community pharmacies.^35^^–^^38^ For example, for the first time in Africa, PrEP is also being implemented in Zambian correctional facilities.^39^ M&E systems will need to adapt within this dynamic landscape.

M&E systems for PrEP need to accommodate new types of PrEP medications, which will impact the frequency and type of clinical services received.

#### Use PrEP Data to Inform Program Implementation

A robust monitoring system for PrEP can provide insight into the PrEP uptake and use across different populations and settings. The usefulness of longitudinal monitoring for PrEP for policymakers, program implementers, and clinicians resides in a country’s ability to use data efficiently to improve PrEP delivery by identifying gaps and implementing strategies responsive to the local context and clients’ needs. PrEP data, combined with other HIV-related data sources, can deliver a more targeted approach to HIV prevention. For example, many countries across SSA are now implementing HIV recency testing, distinguishing recent HIV infection (last 12 months) from long-term infection.^40^^,^^41^ Individuals with recent infections are more likely to have a high viral load, which increases their risk of transmitting the virus to others.^42^ The use of different data sources—including PrEP, recency, and the country’s epidemiological profile—can help when deploying additional interventions to improve PrEP uptake in areas with a high proportion of recent infections. An integrated monitoring system can guide interventions aiming to improve PrEP demand creation, screening, and counseling and improve support to maintain high adherence to PrEP for populations at risk for HIV acquisition.

#### Train Health Care Workers

Effectively monitoring individuals along the PrEP cascade requires health care workers to be knowledgeable and skilled in counseling individuals at risk for HIV on the efficacy of PrEP toward HIV acquisition and the importance of maintaining adherence during periods of high risk. The barrier of stigma deters individuals from accessing PrEP services and threatens the successful scale-up of PrEP services.^43^^–^^45^ Health care worker training should aim to increase provider willingness to promote and normalize PrEP and create a welcoming and friendly environment for clients. In Zambia, training and resources were developed to sensitize health care staff and community health workers to the unique needs of clients, including key populations, and to reiterate the necessity of confidentiality and privacy. The MOH is also reviewing a training package for health care workers to include additional training on PrEP and the National PrEP Program M&E Implementation Guide to optimize its dissemination and use.

## CONCLUSION

Using a collaborative and consensus-based approach, Zambia has developed its first national PrEP M&E implementation guide to standardize guidelines for optimizing the delivery of PrEP services to individuals at risk for HIV infection. The guide builds upon global indicators and incorporates specific indicators to meet the country’s needs and provide a comprehensive overview of the PrEP cascade. The implementation guide developed for the Zambia PrEP program will help to address key questions in PrEP implementation and scale-up, thus improving program quality. Zambia’s experience can inform other countries in SSA as they develop their own national M&E implementation approaches for PrEP.

## Supplementary Material

GHSP-D-22-00396-supplement.pdf

## Notes

**Peer Reviewed**

**First published online:** March 27, 2023.
