# Supplementary material for: Addressing the Need for a Preexposure Prophylaxis Monitoring and Evaluation Implementation Guide: Experience From Zambia
Source: Glob Health Sci Pract. 2023 Apr 28;11(2):e2200396. doi: 10.9745/GHSP-D-22-00396 (PMC10141438; doi:10.9745/GHSP-D-22-00396)
Supplement: GHSP-D-22-00396-supplement.pdf [file GHSP-D-22-00396-supplement.pdf]

THE MINISTRY OF HEALTH

National Pre-Exposure Prophylaxis (PrEP) Program Monitoring & Evaluation Implementation Guide

2022

TABLE OF CONTENTS

Acronyms & Abbreviations ... *4*

Foreword ... *6*

Acknowledgements ... *7*

The Zambian National PrEP Program and Scale-Up ... *8*

Overview ... 8

PrEP Efficacy ... 8

Target Populations for PrEP ... 8

PrEP Scale-Up in Zambia ... 9

Challenges ... 10

Rationale for a National PrEP Program M&E Implementation Guide ... 10

Objectives of the National PrEP M&E Implementation Guide ... 10

Zambian National PrEP M&E Implementation Guide Components ... *11*

Component 1: The PrEP Cascade ... 11

Component 2: Defining National PrEP Target Groups ... 11

2.1 TARGET GROUPS ... 12

1. Key populations ... 12

2. Serodiscordant couples ... 12

3. Pregnant and breastfeeding women at high risk of acquiring HIV ... 12

4. Adolescent girls and young women at a high risk of acquiring HIV ... 13

5. Other high-risk general populations ... 13

2.2 Indicator Target Setting ... 13

Component 3: Routine M&E System for PrEP ... 13

Overview ... 13

Strengthen M&E system to integrate PrEP ... 14

Component 4: PrEP Indicators ... 15

Component 5: Surveillance and Program Data Analysis and Use ... 17

Component 6: Cost-Effectiveness Analysis ... 19

Outcome metrics for PrEP Cascade Evaluation: ... 19

Next Steps and Way Forward ... *25*

Appendices ... *26*

Appendix 1: PrEP Initial Patient Form ... 26

Appendix 2: PrEP Follow Up Form ... 27

Appendix 3: PrEP Non-Clinical Form ... 28

Appendix 4: HIV Prevention Outreach Register ... 29

Appendix 5: HIV Counselling & Testing Register ... 31

Appendix 6: Pre-Exposure Prophylaxis Register ... 32

Appendix 7: Current HIA2 Form (PrEP Component) ... 35

Appendix 8: Proposed PrEP HIA2 Form 36
Appendix 9: Adverse Drug Reaction, Medication Error, and Product Quality Reporting Form 37
Appendix 10: PrEP Performance Indicator Reference Table for Core Indicators. 38
Appendix 11: National PrEP Program Indicator Matrix 49
Appendix 12: Mapping of the Indicators to the PrEP Cascade 54
References *55*

ACRONYMS & ABBREVIATIONS

AGYW	Adolescent girls and young women
AHI	Acute HIV infection
AIDS	Acquired Immune Deficiency Syndrome
ART	Antiretroviral therapy
ARV	Antiretroviral
CDC	U.S. Centers for Disease Control and Prevention
CSO	Civil society organizations
DHIS2	Demographic Health Information System 2.0
DHO	District Health Officer
FP	Family planning
FSW	Female sex worker
FTC	Emtricitabine
GRZ	Government of Republic of Zambia
HIV	Human Immunodeficiency Virus
HMIS	Health Management Information System
IP	Implementing partner
KP	Key population
M&E	Monitoring and evaluation
MOH	Ministry of Health
MSM	Men who have sex with men
NAC	National HIV/AIDS/STI/TB Council
NHSP	National Health Strategic Plan
PBFW	Pregnant and breastfeeding women
PEPFAR	U.S. President's Plan for Emergency AIDS Relief
PHO	Provincial Health Office
PLHIV	People living with HIV
PP	Priority population

PrEP	Pre-exposure prophylaxis
STI	Sexually transmitted infection
TDF	Tenofovir disoproxil fumarate
UNAIDS	Joint United Nations Programme on HIV/AIDS
USAID	U.S. Agency for International Development
VMMC	Voluntary medical male circumcision
WHO	World Health Organization
ZAMPHIA	Zambia Population-based HIV Impact Assessment
ZAMRA	Zambia Medicines Regulatory Authority
ZDHS	Zambia Demographic Health Survey
ZNDC	Zambia National Data Centre

FOREWORD

HIV continues to be a major public health issue in Zambia, leading to government-led programs to prevent HIV, with support from international development agencies, private voluntary organizations and institutions, civil society, and other nongovernmental organizations. All these stakeholders devote resources, time, and energy into the development of cost-effective interventions to slow the spread of HIV.

Prevention of HIV infection has been at the core of the HIV/AIDS response since the inception of the epidemic, where primary prevention, through individual-focused behavioral interventions, was the fundamental strategy adopted. Over the years, it has been noted that no single HIV prevention strategy will suffice to achieve epidemic control. Despite the availability of a widening array of effective HIV prevention tools and a massive scale-up of HIV treatment in recent years, new infections have not decreased sufficiently to achieve epidemic control.

In 2015, recognizing that HIV pre-exposure prophylaxis (PrEP) has potential population-wide benefits, the World Health Organization (WHO) released new guidelines recommending that PrEP should be offered as a choice to people who are at substantial risk of HIV infection as part of a combination HIV prevention program. The Ministry of Health (MOH) included PrEP as an HIV prevention method in the 2016, 2018, and 2020 Consolidated HIV Prevention and Treatment Guidelines.

In 2017, MOH and National HIV/AIDS/STI/TB Council (NAC), in collaboration with implementing partners, embarked on a process to accelerate the provision of PrEP to those at substantial risk of HIV infection by initiating the development of the PrEP Implementation Framework. The Zambia Implementation Framework for Pre-Exposure Prophylaxis was launched in 2018. This PrEP Program M&E Implementation Guide therefore seeks to provide guidance on comprehensive program implementation and management processes, monitoring and evaluation (M&E) systems, and guidance on HIV drug resistance surveillance monitoring within PrEP service delivery being an additional prevention choice for people at substantial risk of HIV infection.

Given the expansion of health services offering PrEP, surveillance, monitoring, and reporting systems need to be implemented alongside PrEP services, and the progress of PrEP implementation should be evaluated periodically. Robust M&E systems promote the effective and safe delivery of PrEP services and ensure that those who would benefit most are provided with the service

Professor Lackson Kasonka
PERMANENT SECRETARY - TECHNICAL SERVICES
MINISTRY OF HEALTH

ACKNOWLEDGEMENTS

The Government of Zambia would like to extend its appreciation and thanks in particular to the following organizations that contributed to the National PrEP Task Force and/or this PrEP M&E Implementation guide.

Centre for Infectious Disease Research in Zambia (CIDRZ)
Ciheb-Zambia
Clinton Health Access Initiative (CHAI)
Joint United Nations Programme on HIV/AIDS (UNAIDS)
Ministry of Health (MOH)
National HIV/AIDS/STI/TB Council (NAC)
President's Emergency Plan for AIDS Relief (PEPFAR)
Procurement & Supply Management (USAID | PSM GHSC)
World Health Organization (WHO)
U.S. Agency for International Development (USAID)
U.S. Centers for Disease Control and Prevention (CDC)
University of Maryland, Baltimore (UMB)
USAID DISCOVER-Health

Special thanks to the editorial committee:

Ministry of Health (MOH)
Dr. Bupe Musonda
Mr. Peter Funsani
Mr. Mutale Mwango
Mr. Ovost Chooye
Mr. Chimuka Sianyinda
Mr. John Mutukwa
Mr. Munsaka Siamwiza
Mr. Melvin Mwansa
Ms. Jojo Mbonyiwe
Mr. David Ngula

National HIV/AIDS/STI/TB Council (NAC)
Mr. Daliso Mumba
Mrs. Ellen Mubanga

U.S. Centers for Disease Control and Prevention (CDC)
Dr. Jackson Okuku

U.S. Agency for International Development (USAID)
Ms. Heidi O'Bra
Ms. Michelle Kim
Ms. Musonda Musonda
Ms. Erin Berghammer
Mr. Arthur Kachemba

University of Maryland, Baltimore (UMB)
Dr. Cassidy Claassen
Dr. Lottie Hachaambwa
Dr. Robb Sheneberger
Mrs. Linah Kampilimba-Mwango
Mr. Olufunso Adebayo
Mr. Morley Mujansi
Dr. Marie-Claude Lavoie
Dr. Natalia Blanco-Herrera
Ms. Anna Whittington
Dr. Kristen Stafford
Dr. Brianna Lindsay

Boston University
Dr. Brooke Nichols

USAID DISCOVER-Health
Dr. Luigi Cuccio
Mr. Japhet Michelo
Ms. Mercy Chisashi
Ms. Mwansa Njelesani-Kaira

Centre for Infectious Disease Research in Zambia (CIDRZ)
Dr. Michael Katongo

World Health Organization (WHO)
Dr. Lastone Chitembo

Dr Connie Osbourne
Director General, National Aids Council

THE ZAMBIAN NATIONAL PrEP PROGRAM AND SCALE-UP

OVERVIEW

Zambia's HIV epidemic is generalized, with a persistently high prevalence of 11%-12.0% and annual incidence of 0.61% among adults 15-59 years old.[1,2] HIV transmission is often concentrated in certain groups, including key populations (KPs) and priority populations (PPs) who face substantial psychosocial and structural barriers to accessing health services and continue to drive epidemic transmission.[3,4] These groups have a higher HIV prevalence compared to the general population and would benefit the most from HIV prevention methods, including biomedical interventions such as pre-exposure prophylaxis (PrEP).[5,6]

PrEP EFFICACY

PrEP is a daily course of antiretroviral drugs (ARVs) taken by HIV-negative people to protect themselves from infection. PrEP effectiveness is highly correlated to adherence,[7-11] with studies showing that PrEP is most effective when adherence is >70%.[12] Therefore, when taken consistently and correctly, PrEP is very effective and reduces the risk of HIV infection to near-zero.[13,14] Daily PrEP with tenofovir disoproxil fumarate (TDF) and emtricitabine (FTC) for the prevention of HIV is a highly effective prevention tool, recommended for persons at substantial risk of HIV infection by the World Health Organization (WHO) since 2015.[15]

TARGET POPULATIONS FOR PrEP

PrEP has the potential to reduce HIV acquisition among at-risk populations, including those who may be unable to negotiate condom usage or who engage in high-risk activities. PrEP can be taken discreetly, offers high rates of protection, and does not require negotiation with partners, unlike barrier methods and microbicide gels.[16,17] PrEP is also associated with few safety risks, and there is limited evidence of behavioral risk compensation (i.e., people do not engage in more risky behavior because they are on PrEP).[12] Importantly, unlike male circumcision and antiretroviral therapy (ART), PrEP is neither a one-time nor a lifelong intervention, and the timing of its use is based on assessment of each client's personal risk. Many PrEP clients are likely to go through "seasons of risk" which require PrEP and lower risk periods which may not.[15,18-21]

PrEP was initially recommended for certain affected KPs at high risk of HIV infection; however, it was then broadened to all PPs. WHO recommends PrEP as a priority for populations with an HIV incidence of 3% or more.[22] According to the National AIDS Strategic Framework 2017-2021, KPs and PPs in Zambia at significant risk for HIV infection include adolescent girls and young women (AGYW), adolescent boys and young men (ABYM), inmates, people who inject drugs (PWID), female sex workers (FSWs), men who have sex with men (MSM), transgender (TG) persons, children, pregnant and breastfeeding women (PBFW), displaced persons, persons with disabilities, and people aged 50 years and older.[20]

In 2016, the Zambia MOH adopted the 2015 WHO guidelines[15] and introduced and scaled-up PrEP as a component of combination HIV prevention services, which also includes counseling, family planning, voluntary male medical circumcision (VMMC), and condom distribution as stated in the National AIDS Strategic Framework 2017-2021.[20] PrEP was first introduced in the 2016 Zambia Consolidated Guidelines for Treatment and Prevention of HIV Infection.[23] The guidelines recommended PrEP be offered to serodiscordant couples and could be considered for others at high-risk of infection. For the updated 2018 ART guidelines, the task force adopted risk-based

criteria for PrEP eligibility included in the Pre-Exposure Prophylaxis Initiation form (Appendix 1) rather than population-specific criteria, and thus further expanded PrEP eligibility in 2018.[24]

Currently, the Zambian guidelines recommend that PrEP should be offered as a part of combination prevention of HIV for HIV-negative persons who are exposed to prolonged and substantial risk of acquiring HIV infection. It should also be administered in a systematic manner as a durable prevention strategy for certain sub-populations as indicated in the national PrEP implementation framework. These sub-populations include: serodiscordant couples, KPs, PBFW, AGYW, ABYM, and other individuals who engage in high-risk sexual activities on a prolonged and regular basis.[25]

Recognizing the need for PrEP scale-up as a key strategy for HIV prevention, the National PrEP Task Force was established in September 2017. The Task Force was composed of key personnel from the MOH, NAC, WHO, UNAIDS, the U.S. President's Emergency Plan for AIDS Relief (PEPFAR), U.S. Agency for International Development (USAID), the U.S. Centers for Disease Control and Prevention (CDC), PEPFAR implementing partners (IPs), and civil society organizations (CSOs). The task force's mandate was to lead policy advocacy and formulate a national PrEP implementation framework to guide PrEP service delivery. The PrEP framework recommends a people-centered approach to PrEP service delivery, guided by public health and human rights. See Figure 1 for a timeline of PrEP policy and scale-up.

Figure 1: Timeline of PrEP policy and implementation in Zambia.

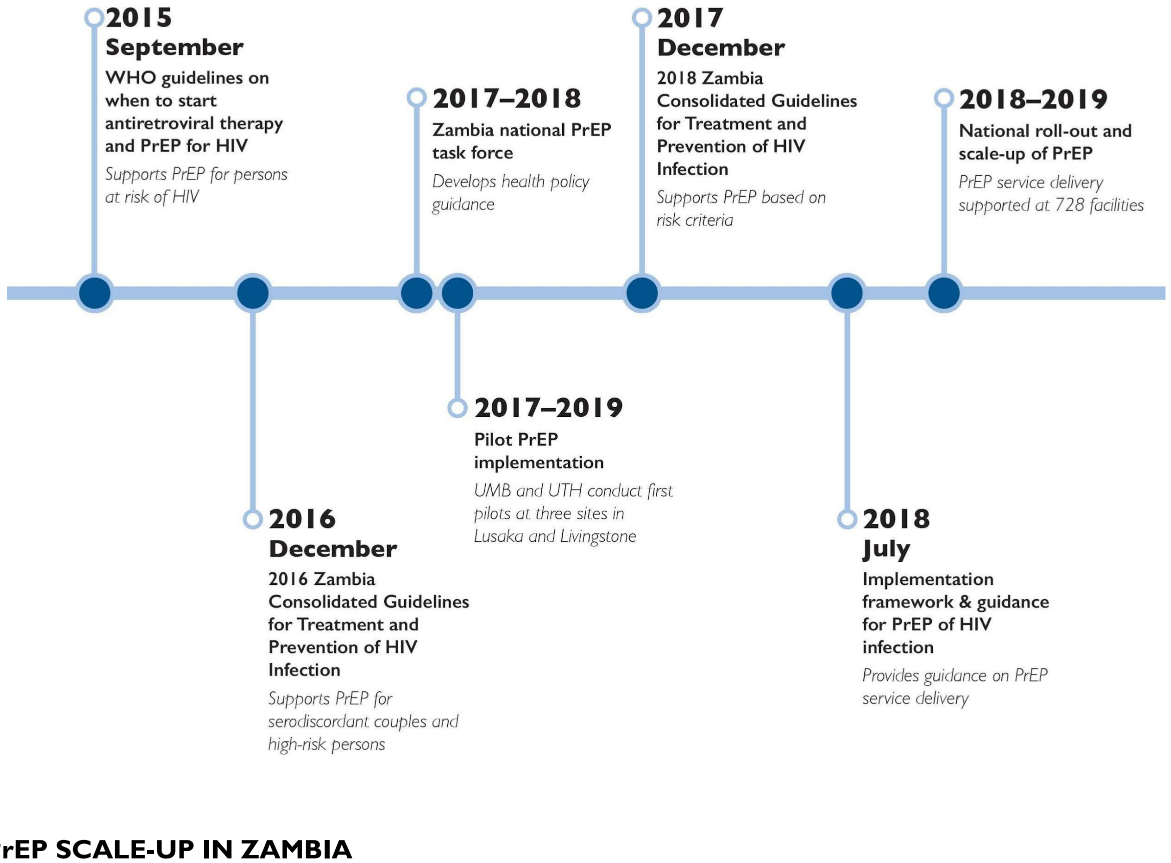

PrEP SCALE-UP IN ZAMBIA

Zambia rolled out PrEP in the public health facilities in 2018. During the first year of PrEP implementation, 3,603 persons had initiated PrEP in two provinces. In year two, the number of people starting PrEP increased more than six-fold to 23,327 persons across 728 sites in all ten Zambian provinces. According to the PEPFAR oversight accountability response team (POART) report of October 2021 showed that by the end of September 2021, a total of 131,260 were enrolled on oral PrEP to prevent HIV infection of which 11% were pregnant and breastfeeding

women, 29% were AGYW and 60% were key populations. The majority of the people that enrolled on PrEP were aged 25+ years. During 2021, there was significant increase in the uptake among adolescent girls and women, of which 45% enrolled via the DREAMS program, attributed to DREAMS services being scaled up to other new districts.

To aid in public education and demand creation, a national HIV prevention campaign called "Zambia Ending AIDS" was developed and implemented in 2019. This campaign had a key focus on providing information on PrEP, as well as education surrounding other evidence-based prevention interventions including condoms, voluntary medical male circumcision (VMMC), family planning (FP), and HIV treatment for HIV-positive individuals.

CHALLENGES

The PrEP program has faced various challenges, both on a public health scale and on the clinical service delivery aspect since its implementation. Generally, these challenges include low demand and limited knowledge among target populations and the public, and barriers to access PrEP at the facility level such as PrEP services being limited to ART clinics and DREAMS sites. These challenges also include low HIV risk perception, stigma, and discrimination, where misconceptions on the use of PrEP and who is eligible to take it still exist. In addition, PrEP monitoring, and evaluation (M&E) operations remain inadequate with the absence of population data (on PrEP targets and indicators). Current targets and indicators are funded-program specific. This gap has been compounded by limited integration and utilization of PrEP data into national MOH systems. Clinical monitoring of PrEP use and continuation remains a challenge as well, particularly due to inadequate resources for the program to operate and insufficient capacity of health workers to conduct proper data analysis and use. It is therefore expedient that measuring PrEP indicators along the PrEP cascade and strengthening the M&E system will assist in identifying newer implementation challenges, deploying solutions, and measuring progress.

RATIONALE FOR A NATIONAL PrEP PROGRAM M&E IMPLEMENTATION GUIDE

The HIV care continuum comprises a series of steps from HIV diagnosis to viral suppression accompanied with well-defined indicators.[26] However, indicators along the PrEP care continuum are in the early stages and still evolving. Hence, having in place a National M&E Implementation Guide for PrEP programs will offer all in-country PrEP service providers the uniform guidance required to measure the availability and delivery of PrEP services systematically and accurately along the cascade. In addition, an M&E system that integrates PrEP data management into the existing country-level HMIS can guide policymakers, program implementers, clinicians, and the community to optimize the coverage, utilization, and safety of PrEP.

OBJECTIVES OF THE NATIONAL PrEP M&E IMPLEMENTATION GUIDE

1. To standardize the monitoring and evaluation of PrEP services.
2. To guide stakeholders on determining PrEP target groups
3. To provide guidance on how to collect and report PrEP indicators along the PrEP cascade.
4. To present cost-effectiveness methodology for PrEP.
5. To promote a data-driven approach to PrEP services delivery for program improvement and decision-making.

ZAMBIAN NATIONAL PrEP M&E IMPLEMENTATION GUIDE COMPONENTS

COMPONENT 1: THE PrEP CASCADE

The PrEP cascade consists of a series of steps to optimize the delivery of PrEP care continuum.[27-29]. In Figure 1, we present the PrEP cascade, which includes (1) identifying people at substantial risk of HIV acquisition through screening, (2) assessing eligibility and willingness for PrEP, (3) initiating the PrEP regimen, (4) adherence, (5) continuation on PrEP at different time points, (6) discontinuing PrEP. [27] Contrary to HIV treatment, which requires a lifelong commitment to ART, HIV-negative clients may discontinue PrEP for various reasons and re-start when needed if eligible. For example, an individual may discontinue PrEP as his/her risk of HIV acquisition has reduced due to a change in sexual behaviors or partners. This "season of risk" must be considered while tracking individuals over-time as they may have discontinued PrEP for different reasons and may re-engage in PrEP care services in subsequent years.

At each step along the PrEP cascade, individuals may drop off. Tracking indicators along the PrEP cascade can highlight gaps in PrEP accessibility, coverage, and delivery across different populations and geographical areas. Program implementers should utilize this data to investigate further about enablers and roadblocks encountered by individuals seeking or obtaining PrEP services to improve the quality and accessibility of PrEP services.

Figure 2: PrEP Cascade.

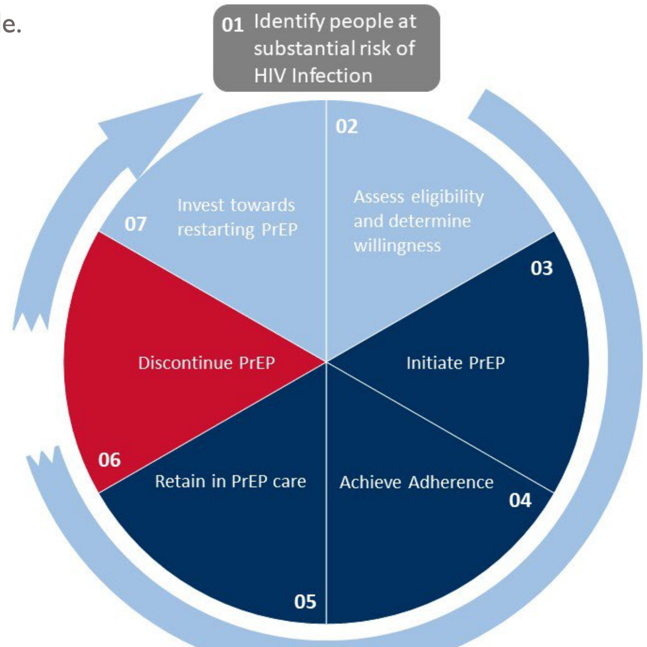

Source: Adapted from Liu et al. and WHO.[27-30]

COMPONENT 2: DEFINING NATIONAL PrEP TARGET GROUPS

To move the country towards epidemic control of HIV, it is imperative to ensure that a concerted effort is directed towards the prevention of new infections amongst those in the HIV-negative population who are substantially at risk of infection.

Therefore, active screening for clients and PrEP implementation scale-up through cost-effective, accessible, and evidence-based targeting will help the national PrEP program to strategize and direct funding as needed to successfully reach those individuals who could most benefit from PrEP services. Screening targets vary based on the relationship between PrEP eligibility and HIV_TST_NEG targets

and should be informed through considerations of achievable PrEP_NEW and PrEP_CT given the sequence of the PrEP cascade.

2.1 TARGET GROUPS

According to the Zambia Implementation Framework & Guidance for Pre-Exposure Prophylaxis of HIV Infection[25] and the World Health Organization,[27] PrEP targets should include the following groups based on their HIV risk:

1. KEY POPULATIONS

As defined by the Zambia National AIDS Strategic Framework 2017-2021,[20] KPs include FSWs, MSM, TG, PWID, and prisoners. KP sizes are not well known in Zambia; however, PEPFAR Zambia undertook a size estimate exercise in 2019, which estimated the MSM population to be 68,044 with an HIV prevalence rate of 17.7% and the population of FSWs to be 133,566 with an HIV prevalence of 41.6%.[31]

The size of these groups can be estimated through surveys such as the Zambia Population-based HIV Impact Assessment (ZAMPHIA), biobehavioral surveys, and triangulation with program data on service use. The proportion of each KP group that is HIV-negative and substantially at risk of infection and who may be reached should be considered in the estimation. However, it is important to note that estimating the size of KPs that should be offered PrEP is challenging due to the stigma and criminalization that members of these communities may face.[32] Therefore, use of program data alone is likely to underestimate the size of the populations in need of PrEP. Empirical methods that can be used to estimate the size of each KP include multiple-source capture-recapture.[33] Methods such as this account for lack of social visibility in the Bayesian methods used for the estimates.[34,35] All methods should consider both venue-based and non-venue settings such as sex workers working on streets or seeking clients online in order to capture all KPs.[36]

2. SERODISCORDANT COUPLES

Serodiscordant couples, in which one partner is HIV-infected and the other is HIV-uninfected, may account for up to half of all new HIV infections in sub-Saharan Africa.[37,38] PrEP can be offered to HIV-negative partners whose partners have not achieved viral suppression on ART. Estimation of the number of serodiscordant couples can be derived from programmatic data, combining the number of new HIV clients starting ART and the number of HIV-positive clients not virally suppressed. ZAMPHIA or similar surveys can also provide information on the proportion of newly HIV-positive individuals in a discordant relationship.

WHO recommends the following formula to estimate this target size: the number of people starting ART multiplied by the probability of being in a discordant relationship. Programs can also be conservative and assume all new clients are in discordant relationships.[27]

3. PREGNANT AND BREASTFEEDING WOMEN AT HIGH RISK OF ACQUIRING HIV

Women are disproportionately affected by HIV in Zambia, representing 58% of adults living with HIV.[39] Several cultural factors lead to this discrepancy, including that some women may not have full control of their sexual and reproductive health, gender-based violence, and limited access to education and income-generating activities.[40]

Targets for pregnant and breastfeeding women at high HIV risk can be estimated using national and program data including census data of women of reproductive age living in high HIV prevalent areas,

local fertility rates, ANC coverage rates, ANC HIV testing rates, and HIV incidence in pregnant women.[27] Similar to other groups at substantial risk for HIV infection, healthcare workers should perform a risk-based assessment included in the PrEP initial form for PBFW (Appendix 1) to determine eligibility for PrEP. Zambia COP20 POART 2021 indicates 14,929 PBFW was initiated on PrEP in COP20 which is 20% of female PrEP_NEW. This was achieved through the integrated service with family planning and Anti natal service provision.

4. ADOLESCENT GIRLS AND YOUNG WOMEN AT A HIGH RISK OF ACQUIRING HIV

Adolescent girls and young women (AGYW) constitute a major priority population. In Zambia, new HIV infections among young women aged 15–24 years were more than double those among young men in 2018.[39] These targets are even more complex but similarly, national and program data can be used to calculate targets. A recommended estimation utilizes the proportion of young girls who have been pregnant or who have been diagnosed with a sexually transmitted infection (STI) multiplied by the number of HIV-negative young girls in a specific geographical region.[27]

5. OTHER HIGH-RISK GENERAL POPULATIONS

In areas with high HIV burden, PrEP interventions among the general population can be cost-effective and should be considered. An early mathematical model of rural Zambia has demonstrated that targeting of high-risk general populations can be considered very cost-effective.[41] An additional study estimated that a five-year PrEP intervention targeting 10% of the HIV-uninfected population in 42 sub-Saharan African countries would lead to a prevention of 390,000 HIV infections.[42] Studies estimate around a 10% PrEP uptake in middle- and low-income countries.[32]

Furthermore, HIV recency data in conjunction with geographic data can be used to identify hotspots or clusters of recent infection where efforts of delivery of PrEP to HIV-negative individuals at high HIV risk are needed.[43]

2.2 INDICATOR TARGET SETTING

All key populations and serodiscordant couples will be considered as potential part of the national target for PrEP. However, amongst the remaining population, mathematical models will be used to estimate targets for PrEP within the population distribution. Uptake of PrEP services amongst these sub population group will be measured alongside the estimates to determine if screening for PrEP and service coverage within each sub population is optimal.

COMPONENT 3: ROUTINE M&E SYSTEM FOR PrEP

OVERVIEW

Zambia uses both paper and electronic systems to collect data at service delivery level. Since 2010, Zambia has had a national electronic health record (EHR) system known as SmartCare (SC). Patient files are recorded at any given clinical visit. Data is entered directly into SC or first using paper-based forms and then entered into SC. The system is primarily designed and used for clinical management. The PrEP Initial (Appendix 1) and PrEP Follow Up (Appendix 2) forms are included as part of the PrEP module in SC. This EMR is rapidly evolving, however, gaps persist related to the quality of the data and restricted functionality of SC to be utilized for public health and programmatic purposes.[44]

For aggregate data, health facilities submit the Service Delivery Reporting Form, also known as the Health Information Aggregation (HIA), every month. This form includes aggregate data on different

health topics, including HIV prevention, care, and treatment. Data are collected from various sources including SmartCare, paper-based registers, and forms. The health facility submits the Service Delivery Reporting Form to the District Health Office where data is entered into DHIS2 before submitting it to the provincial level. Data analysis and verification is conducted at every level.

STRENGTHEN M&E SYSTEM TO INTEGRATE PrEP

Policymakers, program implementers, and clinicians will utilize routinely collected data to improve service delivery by identifying underserved populations, detecting side effects promptly, estimating the cost, and forecasting the demand for PrEP to prevent stock out and ensure adequate financing. This data assists in understanding how PrEP can avert new HIV infections. PrEP data can also be triangulated with other data sources to deploy more effective HIV prevention strategies. For example, the new technology point-of-care recency tests can detect recent HIV infection. PrEP combined with recency data can highlight geographical areas requiring additional HIV prevention strategies, including an increase in the coverage and uptake of PrEP.

To optimize the collection, reporting, and utilization of PrEP and fill existing gaps, the existing M&E system should be strengthened by the following:

1. **Expanding the national health data repository at Infratel.** Currently, the National HMIS Database (DHIS2) and SmartCare EHR platforms have their repositories hosted at Infratel. To ensure continuous integration of all national health program data systems, PrEP data will flow from the facility, district, province, and finally at national level. PrEP program data at the repository will include both aggregated and client-level data captured from the PrEP care continuum.

2. **Integration of PrEP into the Health Management Information System (HMIS)**. Currently, PrEP has both limited data elements and disaggregation (age, sex, and population sub-groups) being collected on the HIA forms. To optimize the integration of PrEP reporting within the national system, a subsection on PrEP can be added to the HIV prevention, care, and treatment section of the HIA2 to capture the number of new and existing clients on PrEP (PrEP_NEW and PrEP_CT). Appendix 7 is the proposed addendum to HIA2 form for PrEP Indicators.

3. **Continue to strengthen the data quality assurance system for the client-level data in SmartCare and DHIS2 Tracker** by conducting regular data quality assessments, data reviews, training, and supervision in collaboration with the PHOs, DHOs and Strategic Information Teams, thereby making room for improving national PrEP program data quality.

As illustrated in Figure 3.3 below, we recommend closer integration with the national health data repository at Infratel as the central repository data warehouse for all PrEP data generated by all implementing partners in Zambia. The architecture of the data warehouse will allow for a data exchange process from the longitudinal tracking system established by all partners. The functional data exchanges will allow for bringing together unduplicated aggregate client-level PrEP data. While the proposed M&E system unifies the PrEP data at the national and sub-national levels, analysis of the data will still need to be performed across all levels to optimize data utilization to track performances, as well as identify gaps and best practices. However, data derived from health facilities or community-based PrEP programs will remain at that level as the primary data sources.

The implementation of PrEP in Zambia is still evolving. Therefore, the M&E system should be agile and flexible to accommodate evolving needs and PrEP service delivery models. Currently, PrEP services are offered both at the community and facility level requiring a standardized and integrated system

that can follow the clients independently of the location of PrEP services to prevent disjointed care and duplication of data. At community and facility level, a platform such as DHIS2 Tracker[45] which enables the collection and analysis of individual level data will assist in tracking individuals across community and facility settings. To achieve this objective, strong data security and unique identifiers are required. At all times client confidentiality must be maintained. In Zambia, the health sector approach to data is the Triple-A approach whereby all levels are involved in assessing, analyzing, and acting on the data.

Figure 3: Recommended data flow for the national PrEP M&E system for both community- and facility-based implementers.

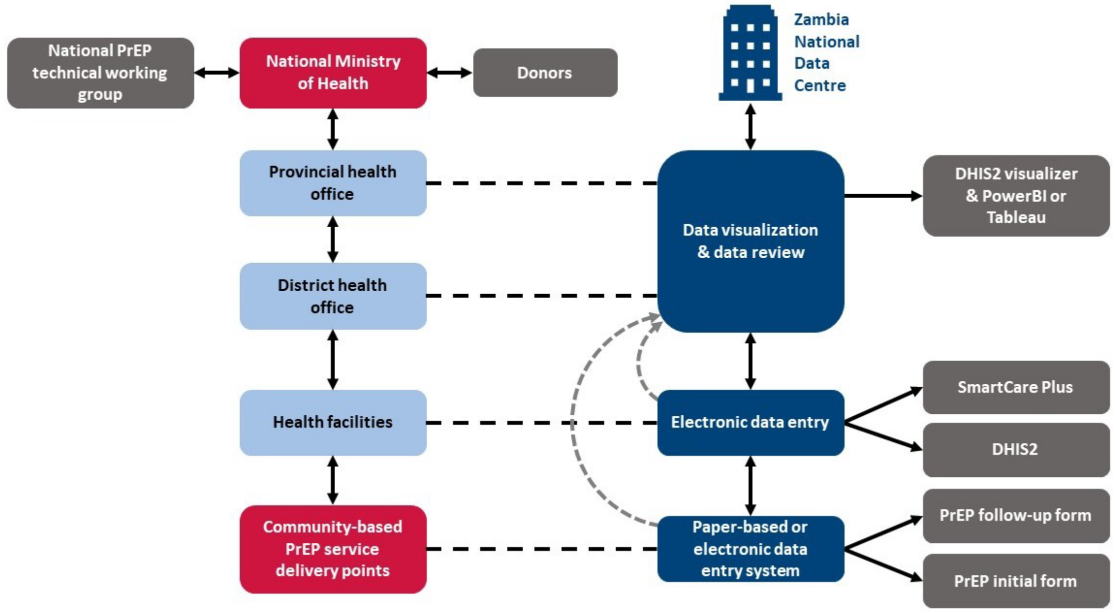

COMPONENT 4: PrEP INDICATORS

This PrEP M&E implementation guide proposes set of core indicators to track individuals along the PrEP cascade to optimize PrEP service delivery. Adapted from the WHO Implementation Tool for PrEP of HIV Infection,[27] the Zambia M&E implementation guide recommends a set of core PrEP indicators (Table 1). Inclusion of these new PrEP indicators may require additional adjustments to the existing M&E system and data collection tools for PrEP. Appendix 9 contains the reference sheet for the proposed core PrEP indicators.

Data should be disaggregated at different levels including age groups, sex, KP, geographical and administrative areas. For KPs, if a person identifies as belonging to more than one KP (e.g., FSW, who inject drugs), the self-reported dominant KP group should be recorded to avoid double counting. The sum of the data disaggregated by type of sub-populations cannot, therefore, be greater than the total.

With the exception of PrEP demand creation activities at both community and health facilities level, data officers and clerks capture PrEP data either with the MOH-approved PrEP Initial H&P or PrEP Follow-Up forms (Appendices 1 & 2). Client-level data is entered in SC while aggregate data is entered into DHIS2.

For PrEP demand creation at the community level, this PrEP M&E Implementation Guide recommends using the PrEP Non-Clinical Form (Appendix 3) for the initial substantial risk assessment while the HIV Prevention Outreach Register (Appendix 4) used to document and track the aggregate level of data on the number of HIV negative individuals who are at substantial at HIV risk referred for PrEP screening and services (PrEP_REFER). However, at health facility level, this guide recommends the use of MOH HIV Counselling and Testing Register (Appendix 5) to document and count this same PrEP demand creation element. This data should be captured in DHIS2.

TABLE 1: CORE PrEP INDICATORS

TYPE OF INDICATOR	INDICATOR	INDICATOR ABBREVIATION	DESCRIPTION	DATA SOURCES
Process	PrEP demand creation activities at community and Health facility level	PrEP_REFER	Number of HIV negative individuals referred for PrEP screening	- PrEP non-clinical form<br>- HIV prevention outreach register<br>- HIV Counselling and Testing Register
Output	Individuals screened for PrEP	PrEP_SCREEN	Number of HIV negative individuals screened for PrEP services.	- PrEP initial form<br>- SmartCare
Output/ Coverage	PrEP Screening Coverage	PrEP_SCREEN_ COV	Percentage of HIV negative individuals screened for PrEP services of the target group (estimated)	- PrEP initial form<br>- SmartCare<br>-Denominator will be obtained using modeling
Output/ Coverage	PrEP Eligibility	PrEP_ELIGIB	Percentage of screened individuals who are eligible for PrEP services.	- PrEP initial form<br>- SmartCare
Output/Service Coverage	PrEP Uptake*	PrEP_UP	Percentage of eligible people who were initiated on antiretroviral PrEP.	- PrEP initial form<br>- SmartCare
Outcome	Early continuation of PrEP*	PrEP_EARLY	Percentage of individuals who continued on PrEP for three consecutive months post initiation	- PrEP initial form<br>- SmartCare
Patient Safety	Toxicity prevalence*	PrEP_TOX	Percentage of people who received PrEP who have discontinued or interrupted PrEP within a three-month period due to a serious ARV-associated toxicity.	- Adverse drug reaction, medication error, and product quality reporting form<br>- SmartCare
Outcome	HIV positivity*	PrEP_HIVPOS	Percentage of people who test HIV-positive among people who received PrEP at least once in the reporting period and had at least one follow-up HIV test within the reporting period.	- PrEP initial form<br>- SmartCare
Output	Individuals newly on PrEP†	PrEP_NEW	Number of individuals who were newly enrolled on antiretroviral PrEP	- PrEP initial form<br>- PrEP register<br>- SmartCare
Outcome	Individuals on PrEP and continuing or restarting PrEP†	PrEP_CT	Number of individuals that returned for a follow-up or re-initiation visit to receive PrEP during the reporting period	- PrEP Follow-up form<br>- PrEP register<br>- SmartCare
Outcome	PrEP continuation	PrEP_CONT	Percent of individuals active on PrEP (6, 9, 12 and >12 months).	- PrEP follow up form<br>- SmartCare
Outcome	PrEP discontinuation and reasons	PrEP_DISC	Percentage of individuals discontinued on PrEP	- PrEP follow up form<br>- SmartCare
Frequency: recommended Monthly, Quarterly, Semi-annually, and Annually.				

* Indicators were adapted from the WHO Implementation tool for PrEP of HIV Infection. [27]
† Indicators from the PEPFAR Monitoring, Evaluation and Reporting Indicator Reference Guide. [46]

COMPONENT 5: SURVEILLANCE AND PROGRAM DATA ANALYSIS AND USE

HIV Drug Resistance (HIVDR) Surveillance amongst PrEP users

The MOH has established a strong and robust HIV case-based surveillance system for Zambia. This system allows for documentation and tracking of clinical records of any individual (HIV positives on ART and HIV negatives on PrEP) in a way that allows for the addition of care and treatment events and outcomes over time covering the entire HIV prevention, care and treatment cascade. In addition, the country has adopted appropriate 2020 WHO guidelines that informed the design and implementation of the HIV drug resistance (HIVDR) surveillance system for both pretreatment and acquired HIVDR. With Zambia scaling up PrEP services, it is essential to establish a system for following up on all HIV sero-converted clients on PrEP. HIV prevention in the form of PrEP scale-up should always be accompanied by measures to monitor and improve the quality of ART delivery. Furthermore, surveillance of HIVDR should include the surveillance of pretreatment HIVDR in populations initiating ART for prevention .

Program Data Analysis and Use

To ensure that the Zambian PrEP scale-up program is data driven at all levels (i.e., National, Provincial, Districts and Site level - both at health facility and community), it is essential for all in-country PrEP implementing partners (IPs) to design and establish a data demand, analysis, and data use plan. This plan must seek to obtain and utilize (at appropriate level) all relevant information obtained from the routine PrEP program data that's coming through the collection and reporting on all indicators agreed and described above in this document. Table 2 below provides a generic guidance on priority level of data analysis and data use for PrEP program at all levels and the critical programmatic decisions and potential evaluation questions that can be deduced for each indicator.

At various level (i.e., national, sub-national, and IP level), the relevance and priority level attached to each indicator differs with respect to the level of analysis and use in making programmatic decisions, effecting policy recommendations and the type of evaluation questions that can be answered. To summarize this information in the priority matrix below, we considered 3 priority levels – extreme, high, and low – as depicted by the legend below.

Table 2: Data Analysis and Data Use Priority Matrix for National PrEP M&E Indicators

Indicator Code	Level of relevance and priority of the National PrEP M&E Indicators to each level (with respect to Data Analysis and Use)					Critical PrEP program decisions and evaluation questions that can be deduced from each indicator
	National level	Provincial level	District level	Health Facility level	Community level	
**PrEP_REFER**	√	√	√	√√√	√√√	• Is the IP creating demand for PrEP amongst HIV negative target groups at both community and health facility level and referring them for full PrEP screening?
**PrEP_SCREEN**	√	√	√	√√√	√√√	• Is the health facility and community mobilization and PrEP recruitment efforts for PrEP target groups yielding good numbers?

Indicator Code	Level of relevance and priority of the National PrEP M&E Indicators to each level (with respect to Data Analysis and Use)					Critical PrEP program decisions and evaluation questions that can be deduced from each indicator
	National level	Provincial level	District level	Health Facility level	Community level	
**PrEP_SCREEN_COV**	√√	√√	√√	√	√√√	• Is the IP and other levels (National and Sub national) covering the relevant and estimated target groups by assessing their needs for PrEP interventions?
**PrEP_ELIGIB**	√√√	√√√	√√√	√√√	√√√	• Does the IP and other levels (National and Sub national) have available adequate quantities of relevant commodities to meet the PrEP need?<br>• What number of PrEP users (and commodities) do we plan for based on the routine data we are getting from the program?
**PrEP_UP**	√	√	√	√√√	√√√	• What are the barriers to low PrEP uptake rates and what can be done to improve it?<br>• Are the PrEP uptake rates declining or improving amongst the respective target groups and what major shifts should be made?
**PrEP_NEW**	√√√	√√√	√√√	√√√	√√√	• Is the number of new PrEP adopters increasing at the rate at which all level (National and Sub national) will meet the annual assigned targets for each subgroups?
**PrEP_CT**	√	√	√	√√√	√√√	• What is the mean duration of different target group on PrEP?<br>• Are the most-at-risk target groups using PrEP long enough?
**PrEP_EARLY**	√	√	√	√√√	√√√	• What is the response like for each target group with respect to early retention and continuation on PrEP?
**PrEP_TOX**	√√√	√√√	√√√	√√	√√	• What is the safety level of PrEP use amongst PrEP user on early retention track?<br>• What are the implicating ARVs and product safety issues to be addressed at policy level?
**PrEP_HIVPOS**	√	√	√	√√√	√√√	• What is the HIV sero-conversion pattern and HIV Incidence amongst the various target groups and what should be done to improve it?
**PrEP_CONT**	√	√	√	√√√	√√√	• What is the mean duration of different target group on PrEP?<br>• Are the most-at-risk target groups using PrEP long enough?
**PrEP_DISC**	√	√	√	√√√	√√√	• What are the critical factors responsible for discontinuation on PrEP services?

Legend: √√√- Extreme Priority, √√– High Priority and √ – Low Priority.

COMPONENT 6: COST-EFFECTIVENESS ANALYSIS

There are several key components to PrEP programming along the PrEP care cascade, each with a different expected effectiveness measure. These metrics are all intended to compare at least two programs that intend to achieve the same outcome metric in each population. Once the evidence base grows and multiple rounds of evaluation have been completed, program outcome and cost-effectiveness benchmarks can be developed.

Evaluation of PrEP programming should be based on time-dependent cohorts of individuals initiating PrEP. For example, the denominator of 12-month retention on PrEP should only contain individuals that would have had the opportunity to provide a 12-month outcome and the costs associated with those individuals. For each of the cost and effectiveness metrics described in this section, we are firstly assuming 12 months of costs and 12 months of outcomes. Should a shorter time period be desired, all of the costs and outcomes should reflect that time period for each program being assessed and compared.

Visual comparison

First, programs should visualize their PrEP cascades for a given risk group that is being targeted. This should show the cascade from PrEP_SCREEN, to PrEP_ELIGB, to PrEP_NEW, to the derived PrEP_SUCCESS indicator described below and based on the indicators above.

OUTCOME METRICS FOR PrEP CASCADE EVALUATION:

Effectiveness Measure, Step 1: *Offer of PrEP*

To evaluate the effectiveness of demand creation programs among providers, the key indicator used in this metric is PrEP_SCREEN_COV, or the proportion of people screened for PrEP services divided by the number of HIV negative individuals due for screening. Since the uptake of a PrEP offer is also further dependent on demand creation in the community, in addition to among the providers, that will be captured in the evaluation of Step 2.

The effectiveness measure for this first indicator ($\text{Effectiveness}_{\text{assess}}$) should therefore be across a specified time period (in this instance, 12 months), and simply reflect the indicator PrEP_SCREEN_COV directly.

This simple effectiveness measure evaluates the complete first part of the cascade.

Cost of Step 1

Here, we focus on the costs related to demand creation and training of providers that are expected to offer PrEP. To measure this step in the cascade, the total costs involved in provider training and mentoring should be included, as well as the cost per person assessed, which includes: provider time with each patient assessed for PrEP, facility equipment, consumables, and program and facility overhead.

Typically, investments in training and mentoring have a lifespan of two years. Therefore, these costs should be annualized when considering the cost per person assessed for PrEP and divided amongst all those assessed for PrEP over a one-year time horizon.

The total cost of Program X across a time period of 12 months would therefore be:

$$\left(\frac{Total\ cost\ of\ training\ and\ mentoring_{12}}{PrEP_{SCREEN_{12}}}\right) + (PrEP_{SCREEN_{12}} * Cost_{PersonAssessed})$$

Cost effectiveness metric for Step 1:

To assess the cost effectiveness of this step, we will look at the additional cost per additional percentage point of people that start PrEP:

$$\frac{Cost_{ASSESS}\ Program\ X_{12} - Cost_{ASSESS}\ Program\ Y_{12}}{Effectiveness_{ASSESS}\ Program\ X_{12} - Effectiveness_{ASSESS}\ Program\ Y_{12}}$$

Effectiveness Measure, Step 2: PrEP Uptake

The individual uptake of PrEP, upon offer of PrEP, is likely a combination of 1) personal perceived risk, 2) knowledge of provider, and 3) demand creation in the community. Because the perceived risk of acquiring HIV infection is likely to differ between different PPs, this effectiveness measure should only compare programs that target the same risk group.

Therefore, this effectiveness measure will be a combination of the indicators "PrEP_ELIGB" (number of individuals eligible for PrEP), and "PrEP_NEW" (number of people initiating on PrEP). Extensive mobilization of the population for PrEP would likely be reflected in the uptake of PrEP among those who are offered and eligible for PrEP.

Therefore, the effectiveness measure across a twelve-month time period for this indicator ($Effectiveness_{uptake}$) should be:

$$\frac{PrEP_{NEW_{12}}}{PrEP_{ELIGB_{12}}}$$

Cost of Step 2

Given that the uptake of PrEP is likely due to a combination of demand creation in the community, the costs associated with that demand creation will be captured here. To measure this step in the cascade, the total costs involved in demand creation should be considered. For example, this could include costs associated with leaflet design, printing, and distribution; cost creating and airing radio or television messaging; time going door-to-door or hosting community events, etc.

For any widescale demand creation program, the costs should be carefully apportioned to just the proportion of the population that a given PrEP program is targeting. For example, we would not want to assign the total cost of a national radio demand creation program only to people who initiate PrEP in Luapula Province. The total cost of these demand creation programs should then be divided by the total number of people that initiate PrEP in a 12-month window.

The cost of the PrEP initiation visit itself will be captured in the third metric where the success of programs in supporting clients on PrEP is evaluated.

Cost effectiveness metric for Step 2:

To assess the cost effectiveness of this step, we will look at the additional cost per additional percentage point of people that initiate PrEP:

$$\frac{Cost_{UPTAKE}\ Program\ X_{12} - Cost_{UPTAKE} Program\ Y_{12}}{Effectiveness_{UPTAKE}\ Program\ X_{12} - Effectiveness_{UPTAKE}\ Program\ Y_{12}}$$

***Effectiveness Measure, Step 3*:** Client support post-initiation, adapted from Hendrickson et al.[19]

Defining PrEP success

Unlike male circumcision and ART, PrEP is neither a one-time nor a lifelong intervention, and the timing of its use is based on assessment of each client's personal risk. Many PrEP clients are likely to go through "seasons of risk" which require PrEP and lower risk periods which may not.[15,18-21]

Seasons of risk may include, for example, the end of a long-term relationship, the beginning of a new sexual practice, immigration to a new city, unanticipated economic hardship, seasonal engagement in sex work, or going on vacation. Changes in risk behavior can go in either direction, portending a time of risk when PrEP is necessary or ending a time of risk when PrEP is no longer necessary. It is thus illogical to count all of those who come off PrEP as a non-successful outcome, as many no longer require it due to lowered risk of HIV acquisition. As a result, healthcare workers must either assess potential risk during prevention counseling with the patient or use formal risk assessments. Expecting near perfect PrEP adherence regardless of risk status is impractical. Therefore, a meaningful metric for PrEP success must incorporate a measure of "persistence on PrEP during periods of risk" to consider behavior change and increased or decreased risk of HIV acquisition over an individual's life.

In Zambia, the definition and determination of risk is based on a risk evaluation tool. This metric assumes that this risk evaluation suitably identifies patients at "substantial risk" of infection.

Once an individual client's risk level is considered, a metric for evaluating PrEP success must also consider two unsuccessful use scenarios. First, if a PrEP client drops out of care without any recorded consultation with a healthcare provider and a re-assessment of risk, it cannot be considered a success, as the reason for drop-out is unclear and may not reflect a reduction in risk. Second, if seroconversion occurs despite the use, or prescription of, PrEP, it is clearly not a success.

Metric inputs

As explained above, the proposed metric should allow for the comparison of programs targeting similar population groups. For example, an FSW program may appear to be less successful than a program for MSM, but this is not reason to cut all programs reaching FSWs in favor of those for MSM. Rather, this effectiveness measure should evaluate programs within risk groups.

We thus define a successful PrEP outcome as a person who initiates PrEP and 1) does not seroconvert; 2) does not have more than one follow-up period at substantial risk of HIV infection but not on PrEP; and, 3) remains engaged in care either by attending visits or discontinuing PrEP use in consultation with a healthcare provider, all within a stipulated follow-up period from PrEP initiation (e.g. 12 months).

Estimating individual PrEP client scores

For each PrEP client at each scheduled follow-up visit, we propose to estimate a score using routinely collected data to answer the questions in Table 2 and convert them to client scores. While Table 2 can be applied to any time period, we suggest that a reasonable initial period of analysis is 12 months. Previously, different timeframes have been used by various service delivery models, making it difficult to compare across programs. We propose that a standard 12-month time period be used for this metric, thereby further improving compatibility of the metric and allowing cross-program evaluation. We propose that this time-period start at a client's date of PrEP initiation. The final row in Table 2, the PrEP success score, incorporates all the data and criteria described earlier in the table. We note that this metric is binary to avoid weighting unsuccessful outcomes without evidence of how to do this. A PrEP client who seroconverts and a PrEP client who is lost to follow-up is each considered unsuccessful. We also note that Table 2 applies only to clients who have already started PrEP—it says nothing about uptake of PrEP among those who have not started but are considered at substantial risk

TABLE 2: INPUTS, OUTPUTS, AND FORMULAE FOR THE GENERATION OF A PROGRAM'S "PrEP SUCCESS OUTCOME"

INDIVIDUAL VISIT SCORE

MEASURED INDICATOR	OUTPUT SCORE	USED FOR
**PrEP_EARLY (3 months); PrEP_CONT (6,9,12 months)**	Yes=1, no=0. If a PrEP client does not miss more than one scheduled clinic visit during the 12-month period, then they are considered engaged in care. If they are on PrEP and miss more than one scheduled clinic visit (and the PrEP_DISC reason of 'no longer at risk' was not present), then they are considered to have dropped out of care.	Persistent PrEP use
**PrEP_HIVPOS (b)**	0=negative, 1=positive, or u=unknown.	HIV seroconversion at any point during PrEP follow-up
**PrEP_ELIGB (c)**	Yes=1, no=0	Appropriately on PrEP
**PrEP_DISC (d)**	Yes=1, no=0.	Reason for discontinuing PrEP

FOLLOW-UP PERIOD CALCULATION

SUCCESS CRITERION	CRITERION THRESHOLD
**1. Maintains HIV uninfected status (A)**	If PrEP_HIVPOS= (0) at all clinic visits, then labeled with a success criterion of 1.
**2. PrEP persistence (B)**	If PrEP_EARLY=1 and PrEP_CONT=1 at all time points (or all but one time point), or if PrEP_EARLY=0 or PrEP_CONT=0 and PrEP_DISC reason is "No longer at risk" (e.g. stopping PrEP in consultation with healthcare provider) at all time points (or all but one time point), then labeled with a success criterion of 1.

**3. Appropriately on PrEP* (C)**	If PrEP_ELIGB=1 and PrEP_EARLY/PrEP_CONT=0, then labeled with a success criterion of 0. Otherwise, success criterion=1.	
**OVERALL PrEP SUCCESS SCORE**		
**OUTPUT METRIC**	**INPUT CRITERION**	**FORMULA**
**PrEP success score (i)**	Yes=1; no=0	If A=1, B=1, and C=1, then i=1; else i=0

** "Appropriately on PrEP" depends on a country's PrEP guidelines for determination of risk. PrEP_NEW is the denominator for this metric.*

Estimating program success in supporting PrEP clients

Table 2 explains how to determine if any individual client is a PrEP success. To evaluate a program, rather than an individual client, the number of "PrEP successes" for a group of clients over a specified time period can be divided by the total number of PrEP_NEW in the group during that time period ("$\text{Effectiveness}_{\text{support}}$"). This success ratio can then be used as a meaningful way to compare PrEP strategies across different sites and programs, possibly even at the national level or across countries, depending on differences in definitions of a "PrEP success." As mentioned above, however, evidence has already demonstrated that PrEP risk, uptake, persistence of use, and adherence differ by different population group. We thus caution against comparing programs that target different populations.

Cost of Step 3

To determine the cost of ongoing PrEP support and use, this step should focus on a resource-use based costing strategy.

This should therefore include, at the patient level: the number of facility visits made (derived directly from PrEP_EARLY through three months post initiation, and PrEP_CONT for 6 months, 9 months, and 12 months), or healthcare worker interactions, across a 12-month period; the amount of PrEP dispensed (assuming PrEP is dispensed at each visit unless otherwise noted under the PrEP_DISC indicator); number of labs done (where possible). In addition to this, any type of programmatic PrEP support should be captured in the costs here. This includes any personnel such as PrEP navigators, the training of these personnel, any organized support groups (in person or digital), etc. The cost of these support networks should be divided by the number of patient months of PrEP access and assigned to each patient month of use, accordingly.

It should be noted that there are additional costs to PrEP programming, such as monitoring and evaluation and supply chains. It is, however, assumed that these costs will be equal across all programs and will not be the defining metric in the success of a PrEP program. As such, these costs are not included in any of the cost-effectiveness analyses described in Component 5.

Typically, investments in training have a lifespan of two years. Therefore, the costs of any staff training should be annualized.

The total cost of Program X across a time period of 12 months would therefore be:

$$\left(\frac{Total\ cost\ of\ PrEP\ support}{PrEP\ person\ months\ of\ Program\ X}\right) + \left((PrEP_{EARLY} + PrEP_{CONT}) * (Cost_{visit} + Cost_{PrEPDrugs})\right)$$

*The number of labs and the cost of each of those lab tests may also be added, where data exist.

Cost effectiveness metric for Step 3:

To assess the cost effectiveness of this step, we will look at the additional cost per additional person with a successful PrEP outcome as defined within the effectiveness metric of Step 3:

$$\frac{Cost_{Support}\ Program\ X_{12} - Cost_{Support} Program\ Y_{12}}{Effectiveness_{Support}\ Program\ X_{12} - Effectiveness_{Support}\ Program\ Y_{12}}$$

Evaluation of each step in the cascade

Some programs may excel at any one of these aspects of a PrEP program, and a robust evaluation of each step in the cascade will aid in determining best practices to ensure robust and efficient PrEP programing. It is imperative that PrEP programs learn from one another to strengthen each aspect of the PrEP care cascade.

NEXT STEPS AND WAY FORWARD

As Zambia moves from a generalized HIV epidemic into an AIDS-free era, ensuring access to biomedical HIV prevention methods such as PrEP will be critical. Robust M&E of PrEP rollout, uptake, adherence, adverse events, and cost effectiveness will be required to ensure that all persons at risk of HIV have access to PrEP. To this end, it is recommended that all PrEP service providers in Zambia utilize the methods laid out in this framework to ensure a unified national approach.

Based on the methods presented in this document, all PrEP service providers are expected to adopt the minimum required PrEP indicators and report on these regularly. The optional indicators are recommended but not mandatory at this time. All PrEP service providers are expected to have adopted these standards by 1 January 2023.

Increasing the availability and quality of PrEP routine data is essential for program monitoring, planning, and policy decision-making. Data should be routinely used across all levels to design and implement interventions to address existing gaps. Understanding the leaks along the PrEP cascade can assist in deploying targeted interventions. In addition to the on-going monitoring, program evaluations can assist in measuring the effects of the program. Qualitative or mixed method evaluations can also provide insight into barriers, perceptions and needs of the populations seeking PrEP services.

Leadership and management are integral parts of a successful M&E system. The National PrEP Task Force shall continue to provide oversight of the PrEP M&E system. Following initial implementation of these standards, the PrEP Task Force will continue to review this framework and revise as needed.

NAC and MOH, along with the PrEP Task Force, will coordinate an annual report of PrEP provision across all PrEP service providers, to be coordinated in October of each year (in line with the end of the PEPFAR fiscal year). The first report will be issued in October 2023.

Critically, adoption of this M&E framework will enable improved PrEP programming such that those most in need of PrEP services are reached and supported.

APPENDICES

APPENDIX I: PrEP INITIAL PATIENT FORM

Pre - Exposure Prophylaxis Initial

PROVINCE DISTRICT FACILITY CARECARD No Serial No. CHK Digit

PT ID: Date:

Last Name First Name Female Male DOB

Screening for Substantial Risk for HIV infection

Category/Question

1) Currently sexually active AND report any of the below in last six months Have you been sexually active in the last six months? Yes No

- Has had vaginal or anal intercourse without condoms with more than one partner
  - With how many people did you have vaginal or anal sex in the last six months? 1 2 or more
  - Did you use condoms consistently during sex in the last six months? Yes No
- Has a current sex partner with one or more HIV risk
  - Have you had a sex partner in the last six months who: • Is living with HIV? Yes No • Injects drugs? Yes No • Is a sex worker? Yes No • Is a man who has sex with men? Yes No • Is a transgender person? Yes No • Has sex with multiple partners without condoms? Yes No
- Has had a sexually transmitted infection (STI) (based on self-report, lab test, syndromic STI treatment) in the last six months? Yes No
- Has used post-exposure prophylaxis (PEP) following a potential exposure to HIV in the last six months? Yes No

2) Has shared injection material/equipment with other people in the last six months Yes No

3) Has had a sexual partner in the last six months who is HIV positive AND who has not been on effective* HIV treatment

**If partner has been on ART for less than six months, or has inconsistent or unknown adherence*

- Is your partner HIV infected? Yes No Don't know
- Is he/she on ART? Yes No Don't know
- What was the last viral load result? Yes No Don't know

STI SCREENING

- Urethral/Vaginal discharge Yes No
- Lower abdomen pain Yes No
- Genital sores Yes No
- LN swelling Yes No
- Genital pain/itching Yes No

If yes, follows syndromic management for treatment

OBSTETRIC HISTORY

- Are you currently pregnant? Yes No
- LMP
- EDD
- Are you currently breastfeeding? Yes No
- Client screen for CACX? Yes No

Screen for Acute HIV

- Sore throat Yes No
- Fatigue Yes No
- Fever Yes No
- Rash Yes No
- Chills Yes No
- Headache Yes No
- Muscle aches Yes No

PAST MEDICAL HISTORY

Have you ever been diagnosed with the following diseases

- Hepatitis B Yes No
- Tuberculosis Yes No
- Diabetes Yes No
- Kidney disease Yes No
- Hypertension Yes No
- Psychiatric illness Yes No

PHYSICAL EXAM Weight(kg) BP / Temp. °C . BMI .

Heart rate/min Resp. rate General: Pallor Jaundice Edema

Notable Findings:

Genital Exam:

HIV STATUS

- Date of Last Potential HIV Exposure
- Last Date of HIV Test
- HIV Test Results -ve +ve Indeterminate
- Testing Location Facility Community HIV Self-Test

CURRENT MEDICINES

- ATT
- Anti-HBV
- Anti-Hypertensive
- Anti-Psychotics

PREVENTION

- H/O PEP use
- Condom/ Lube Use
- VMMC
- Used PrEP before

ASSESSMENT

PrEP ELIGIBILITY

- HIV Negative
- No Acute HIV Infection symptoms
- Creatinine Clearance >50
- Urinalysis Normal
- Able and Willing to Adhere to Daily PrEP
- Last Potential HIV Exposure More than 6 Weeks Ago
- HIV Negative Test within Last 2 Weeks

PLAN

Client eligible for PrEP Yes No

Start PrEP

PRESCRIPTION

TDF + FTC

Reason for not starting?

- Acute HIV infection
- Adherence counselling
- Suspected Acute HIV Infection
- Creatinine clearance <50mL/min
- Abnormal Urinalysis

INVESTIGATIONS

- HIV
- RPR
- UA
- CrCl
- Gravindex
- HBsAg
- HVS

REFERRALS

- Family Planning
- STI Screening
- VMMC
- Nutrition
- Adherence
- CaCx screening

PrEP Dispensed

1 Month

Next Clinical Appointment

2 weeks 3 Weeks 1 Month

Date of Next Visit

Staff Name Staff Signature

PrEP V 1.1.1

APPENDIX 2: PrEP FOLLOW UP FORM

PrEP FOLLOW UP

Date: D D M M Y Y

PT ID: PROVINCE | DISTRICT | FACILITY | CARECARD No | Serial No. CHK Digit

○ Female ○ Male

Last Name ______ First Name ______

Date of Last Visit: D D M M Y Y (Day Month Year)

Presenting Complaints ○ Yes ○ No

PREVIOUS LAB RESULTS

Test	Result		Date
HIV		Date	
CrCl		Date	
Hepatitis B		Date	
RST		Date	
UA		Date	

SCREEN FOR ACUTE HIV INFECTION

Symptom		
Swollen nodes	○ Yes	○ No
Fever	○ Yes	○ No
Sore throat	○ Yes	○ No
Muscle aches	○ Yes	○ No
Fatigue	○ Yes	○ No
Rash	○ Yes	○ No
Chills	○ Yes	○ No
Headache	○ Yes	○ No

STI SCREENING

Symptom		
Vaginal urethral discharge	○ Yes	○ No
Lower abdomen pain	○ Yes	○ No
Dysuria	○ Yes	○ No
Genital Ulcers	○ Yes	○ No
Dyspareunia	○ Yes	○ No
Chlamydia	○ Yes	○ No
Gonorrhoea	○ Yes	○ No

If yes, follows syndromic management for treatment

OBSTETRIC HISTORY

LMP

EDD

Are you breastfeeding? ○ Yes ○ No

Is child HIV Tested? ○ Yes ○ No

Client screen for CACX? ○ Yes ○ No

PREVENTION

Client counselled on condoms ○ Yes ○ No

Family Planning ○ Yes ○ No

Condoms given ○ Yes ○ No

PHYSICAL EXAM Weight(kg) ___ . ___ BP ___ / ___ Temp. °C ___ . ___ BMI ___ . ___

Heart rate/min ___ Resp. rate ___ General: ☐ Pallor ☐ Jaundice ☐ Edema Notable Findings: ______

Genital Exam: ______

RISK STATUS

Client wants to continue PrEP ○ Yes ○ No

Reasons for not continuing PrEP

☐ Partner on ART VL suppressed

☐ No longer involved in unsafe practices

☐ Client has one consistent sexual partner

SCREENING FOR SIDE EFFECTS OF TDF/FTC

Symptom			
Headache	○ Yes	○ No	Other:______
Oliguria	○ Yes	○ No	
Nausea	○ Yes	○ No	
Rash	○ Yes	○ No	
Vomiting	○ Yes	○ No	
Numbness	○ Yes	○ No	

ADHERENCE

Problems taking medication

Does client have trouble taking pills ○ Never ○ Rare ○ Sometimes ○ Often

Reasons for missing

☐ Forgot ☐ Side effects ☐ Away from home

☐ Illness ☐ Others ☐ Meds finished

How many doses missed since last pick up?

☐ 0 → Follow regular pharmacy

☐ 1 → Monthly Pharmacy Schedule

☐ ≥2 → Follow 4 wks weekly Appointment

ASSESSMENT

______

PLAN ○ Continue PrEP ○ Stop PrEP

Reasons for stopping

○ Sero conversion to +ve

○ No Longer at Risk ○ Poor Adherence

○ Contraindication to PrEP: ______

INVESTIGATIONS

☐ HIV ☐ PRP

☐ Crcl ☐ Gravindex

☐ HBsAg ☐ HVS

☐ UA ☐ Other:______

PRESCRIPTION

○ TDF + FTC

○ Others ______

○ Others ______

○ Others ______

REFERRALS

☐ Condom lubes ☐ Psychosocial support

☐ Risk Reduction Counselling ☐ CaCx Screening

☐ Partner Referral ☐ VMMC ☐ FP

☐ Adherence Counselling ☐ ART if Sero-Converted

______ Staff Name ______ Staff Signature

PrEP DISPENSED

○ 1 Month ○ 2 Months ○ 3 Months

Next Pharmacy Appointment

○ 1 Month ○ 2 Months ○ 3 Months

Next Clinical Appointment

○ 2 weeks ○ 3 Weeks ○ 1 Month ○ 3 Months ○ Other

Date of Next Visit D D M M Y Y

APPENDIX 3: PrEP NON-CLINICAL FORM

NON-CLINICAL FIRST ENCOUNTER PRE-EXPOSURE PROPHYLAXIS (PrEP) & HIV PREVENTION SCREENING TOOL

Facility Name:	Facility Code:
Provider/ CHW Name:	Client Encounter No.
Client Phone No.	Date:

Screening questions				
No.	Questions	Variables		
1	Gender	Male	Female	
2	How old are you?	years		
3	Where do you live?			
4	Are you trying to conceive a child with a partner who is HIV-positive?	Yes**	No	
5a.	Do you have a sexual partner who has HIV?	Yes*	No	Don't Know*
b.	If "Yes," have they been taking their ARVS without stopping for at least six months?	Yes	No**	Don't Know*
In the past 6 months:				
6	Did you have a sexually transmitted infection? (such as sored or discharge from vagina or penis)	Yes *	No	
7	Did you have sex without a condom with someone who you know hashed or whose HIV status you do not know?	Yes**	No	
8	Were you forced or pressured to have sex?	Yes**	No	
9	Did you use Post-Exposure Prophylaxis (PEP)?	Yes*	No	

Guidance on assessing if a client is a good candidate for PrEP and other HIV prevention services

- **Clients with two or more** *may be good candidates for PrEP. Facilitate referral to the closest service delivery point *with referral form*
- Discuss other prevention options (condoms, risk reduction; VMMC) with each client; regardless of result

APPENDIX 4: HIV PREVENTION OUTREACH REGISTER

Month-Year eg. June 2019

							SERVICES PROVIDED															
							Number of Materials provided				Communication Topics (Tick X all that apply)											
No.	Date of Outreach (dd/mm/yyyy)	CLIENT INFORMATION		Encounter (visit) Number	Date of Visits (dd/mm/yyyy)	Hotspot (Insert the Name)	IEC	Male Condoms	Female Condoms	Lubricant	STI	GBV	PrEP/ PEP	HIV Testing	ART	FP	TB	Substance Use	Cervical Cancer Screening	Prostate Cancer Screening	VMMC	Screening Modality (Questionnaire)
					(a)	(b)	(c)	(d)	(e)	(f)	(g)	(h)	(i)	(j)	(k)	(l)	(m)	(n)	(o)	(p)	(q)	(r)
		Alias/ Nick Name:	Key Population Code:	1																		
		Unique ID Code:	Priority Population Code:	2																		
		Age:		3																		
		Gender Code:		4																		
		Alias/ Nick Name:	Key Population Code:	1																		
		Unique ID Code:	Priority Population Code:	2																		
		Age:		3																		
		Gender Code:		4																		
		Alias/ Nick Name:	Key Population Code:	1																		
		Unique ID Code:	Priority Population Code:	2																		
		Age:		3																		
		Gender Code:		4																		
		Alias/ Nick Name:	Key Population Code:	1																		
		Unique ID Code:	Priority Population Code:	2																		
		Age:		3																		
		Gender Code:		4																		
		Alias/ Nick Name:	Key Population Code:	1																		
		Unique ID Code:	Priority Population Code:	2																		
		Age:		3																		
		Gender Code:		4																		
		Alias/ Nick Name:	Key Population Code:	1																		
		Unique ID Code:	Priority Population Code:	2																		
		Age:		3																		
		Gender Code:		4																		
		Alias/ Nick Name:	Key Population Code:	1																		
		Unique ID Code:	Priority Population Code:	2																		
		Age:		3																		
		Gender Code:		4																		
		Alias/ Nick Name:	Key Population Code:	1																		
		Unique ID Code:	Priority Population Code:	2																		
		Age:		3																		
		Gender Code:		4																		
		Alias/ Nick Name:	Key Population Code:	1																		
		Unique ID Code:	Priority Population Code:	2																		
		Age:		3																		
		Gender Code:		4																		
		Alias/ Nick Name:	Key Population Code:	1																		
		Unique ID Code:	Priority Population Code:	2																		
		Age:		3																		
		Gender Code:		4																		

	HTS ASSESSMENT, TESTING & REFERRAL INFORMATION												CLINICAL LINKAGE INFORMATION															
	HTS Need Assessment (Tick X on the only one that applies)						HIV Testing Modality (Mark X)						Clinical Referral/Provision															
Screening Modality (Self-Test)	HIV Testing Non-Eligible (Known HIV-POS_Not On ART)	HIV Testing Non-Eligible (Known HIV-POS_On ART)	HIV Testing Non-Eligible (Recently Tested)	HIV Testing Non-Eligible [illegible]	HIV Testing Eligible [illegible]	HIV Testing Eligible [illegible]	On-site [illegible]	Referred	HIV Testing Modality (insert the exact HTS modality)	Tested less than 3 months ago (Y/ N)	Date of HTS (dd/mmm/yyyy)	Result of HTS (P/N)	ART	PrEP	STI Screen/Treat	TB Screen/Treat	Cervical cancer screening	Prostate Cancer Screening	Diabetes Screening	GBV Referral	Other	None	ART number	PrEP number	Facility Referred to	Final Outcome at 1 year	Name of CHW/ Peer Navigator	Comments
(s)	(t)	(u)	(v)	(w)	(x)	(y)	(z1)	(z2)	(z3)	(aa)	(ab)	(ac)	(ad)	(ae)	(af)	(ag)	(ah)	(ai)	(aj)	(ak)	(al)	(am)	(an)	(ao)	(ap)	(aq)	(ar)	

APPENDIX 5: HIV COUNSELLING & TESTING REGISTER

Ministry of Health

HTS Register - HIR. -----

Client Registration Details									Prior HIV Test Status		Pre-test Counselling and Testing			Post-test Services			Partner HIV Counselling and Testing				Index Testing Services		
HTS Number	Date of Visit (dd/mm/yyyy)	Name / Residential Address	Client's contact details	Age	Sex (M/F)	Number of Biological Children Alive / Number of Biological Children	Marital Status (Code)	Level of Education (Code)	Tested Before (Y/N) / Date of test	Test Result (P/N/I/ND)	Reason for Testing (code) / Self Test(Y/N)	Eligible to test based on Screening Tool (Y/N) / Consent Given (Y/N)	Test result (P/N/U/ND)	Clients Informed of the Results (Y/N)	Recency Testing done (Y/N/NA) / Recent Infection (Y/N)	Referred for ART (Y/N/NA) / ART Number	Came as a Couple (Y/N) / Partner tested(Y/N/NA)	Partner's HTS Number (Refer to (Col a)	Partner Referred for ART (Y/N/NA) / ART Number	Discordant couple (Y/N/NA)	Index Contact Provided (Y/N)	Service Provider	Remarks
(a)	(b)	(c)	(d)	(e)	(f)	(g)	(h)	(i)	(j)	(k)	(l)	(m)	(n)	(o)	(p)	(q)	(r)	(s)	(t)	(u)	(v)	(w)	(x)
					M F																		
					M F																		
					M F																		
					M F																		
					M F																		
					M F																		
					M F																		
					M F																		
					M F																		
					M F																		
					M F																		
					M F																		
					M F																		
					M F																		
					M F																		
					M F																		
					M F																		
					M F																		
					M F																		
					M F																		
					M F																		
					M F																		
					M F																		
					M F																		
					M F																		
					M F																		
					M F																		

APPENDIX 6: PRE-EXPOSURE PROPHYLAXIS REGISTER

Note: Initiation /Start Date for Current PrEP (Indicated Date Format must be applied) | *dd/mm/yyyy*

Serial No.	Date of Visit (dd/mm/yyyy)	Client Name<br>Client PrEP ID	Client Cell No.<br>Next of Kin Cell No.	Age (Years)	Sex (M/F)	**Client Status**<br>New<br>(Y/N)	Re-start<br>(Y/N)	TI<br>(Y/N)	Continuing<br>(Y/N)	**Client Type Upper:** (By Relationship - 1 or 2)<br>**Lower: Client Type (By Category)** (indicate category that applies- 1, 2, or 3)	**Lower: Client Type (By Population type)** (indicate the Population Type - A, B, C)	**PrEP Medication Prescribed**<br>Regimen<br>Daily Oral Medication (Y/N)	Regimen<br>Others (Y/N)	**PrEP History**<br>Duration<br>Regimen	**Baseline Status - Month 0**<br>HIV<br>(P/N)	HBsAg<br>(P/N)	RPR/RST<br>(P/N)	Creatinine<br>(µmol/L)	ALT<br>(U/L)	PrEP Initiation<br>Date of Initiation<br>Regimen	Current PrEP<br>Start Date<br>Regimen	**Status at Month 1 FollowUp Visit (Upper: Indicate date of this Visit)**<br>HIV Re-test Date<br>(P/N)	HBsAg Re-test Date<br>(P/N)	RPR/RST Re-test Date<br>(P/N)	Creatinine Re-test Date<br>(µmol/L)	ALT Re-test Date<br>(U/L)
(a)	(b)	(c)	(d)	(e)	(f)	(g)	(h)	(i)	(j)	(k)	(l)	(m)	(n)	(o)	(p)	(q)	(r)	(s)	(t)	(u)	(v)	(w)	(x)	(y)	(z)	(aa)
																								3/10/2022		
																						10/03/2022 (N)	10/03/2022 (N)	10/03/2022 (N)	10/03/2022 (XX)	10/03/2022 (XX)

PrEP Continuation at 1 month			Status at Month 3 Follow Up Visit (Upper:Indicate date of this Visit)					PrEP Continuation at 3 month			Status at Month 6 Follow Up Visit (Upper:Indicate date of this Visit)					PrEP Continuation at 6 month			Status at Month 9 Follow Up Visit (Upper:Indicate date of this Visit)					PrEP Continuation at 9 month			
ARV Dispensed (Y/N) Regimen Dispensed	Date Stopped	Reasons for Stopping PrEP (1,2,3, 4,5,6)	HIV Re-test Date (P/N)	HBsAg Re-test Date (P/N)	RPR/RST Re-test Date (P/N)	Creatinine Re-test Date (µmol/L)	ALT Re-test Date (U/L)	ARV Dispensed (Y/N) Regimen Dispensed	Date Stopped	Reasons for Stopping PrEP (1,2,3, 4,5,6)	HIV Re-test Date (P/N)	HBsAg Re-test Date (P/N)	RPR/RST Re-test Date (P/N)	Creatinine Re-test Date (µmol/L)	ALT Re-test Date (U/L)	ARV Dispensed (Y/N) Regimen Dispensed	Date Stopped	Reasons for Stopping PrEP (1,2,3, 4,5,6)	HIV Re-test Date (P/N)	HBsAg Re-test Date (P/N)	RPR/RST Re-test Date (P/N)	Creatinine Re-test Date (µmol/L)	ALT Re-test Date (U/L)	ARV Dispensed (Y/N) Regimen Dispensed	Date Stopped	Reasons for Stopping PrEP (1,2,3, 4,5,6)	HIV Re-test Date (P/N)
(ab)	(ac)	(ad)	(ae)	(af)	(ag)	(ah)	(ai)	(aj)	(ak)	(al)	(am)	(an)	(ao)	(ap)	(aq)	(ar)	(as)	(at)	(au)	(av)	(aw)	(ax)	(ay)	(az)	(ba)	(bb)	(bc)

Status at Month 12 Follow Up Visit (Upper: Indicate date of this Visit)				PrEP Continuation at 12 month			Status at Month 12+ Follow Up Visit (Upper: Indicate date of this Visit)					PrEP Continuation at 12+ month			
HBsAg Re-test Date (P/N)	RPR/RST Re-test Date (P/N)	Creatinine Re-test Date (µmol/L)	ALT Re-test Date (U/L)	ARV Dispensed (Y/N) Regimen Dispensed	Date Stopped	Reasons for Stopping PrEP (1,2,3, 4,5,6)	HIV Re-test Date (P/N)	HBsAg Re-test Date (P/N)	RPR/RST Re-test Date (P/N)	Creatinine Re-test Date (µmol/L)	ALT Re-test Date (U/L)	ARV Dispensed (Y/N) Regimen Dispensed	Date Stopped	Reasons for Stopping PrEP (1,2,3, 4,5,6)	Remarks
(bd)	(be)	(bf)	(bg)	(bh)	(bi)	(bj)	(bk)	(bl)	(bm)	(bn)	(bo)	(bp)	(ad)	(ae)	(af)

APPENDIX 7: CURRENT HIA2 FORM (PrEP COMPONENT)

**3.0 HIV Prevention, Care and Treatment**						
**3.6 Pre Exposure Prophylaxis (PrEP) Services**						
**A. PrEP New**		MALE		FEAMLES		TOTAL
10-14 yrs	HIV-000		HIV-000		HIV-000	
15-19 Yrs	HIV-001		HIV-001		HIV-001	
20-24 Yrs	HIV-002		HIV-002		HIV-002	
25-49 Yrs	HIV-003		HIV-003		HIV-003	
50+ Yrs	HIV-004		HIV-004		HIV-004	
Totals	HIV-004		HIV-004		HIV-004	
**b. PrEP CURR**						
		MALES		FEMALES		TOTAL
10-14 yrs	HIV-000		HIV-000		HIV-000	
15-19 Yrs	HIV-001		HIV-001		HIV-001	
20-24 Yrs	HIV-002		HIV-002		HIV-002	
25-49 Yrs	HIV-003		HIV-003		HIV-003	
50+ Yrs	HIV-004		HIV-004		HIV-004	
Totals	HIV-004		HIV-004		HIV-004	
**c. PrEP Population Type**						
PBFW	HIV-000					
Adolescents	HIV-001					
Discordant Couples	HIV-002					
KPs	HIV-003					
Others	HIV-004					
Total PrEP	HIV-005					
**d. PrEP Adverse**						
		**MALES**		**FEMALES**		**TOTAL**
10-14 yrs	HIV-000		HIV-000		HIV-000	
15-19 Yrs	HIV-001		HIV-001		HIV-001	
20-24 Yrs	HIV-002		HIV-002		HIV-002	
25-49 Yrs	HIV-003		HIV-003		HIV-003	
50+ Yrs	HIV-004		HIV-004		HIV-004	
Total	HIV-004		HIV-004		HIV-004	

APPENDIX 8: PROPOSED PrEP HIA2 FORM

3.0 HIV Prevention, Care and Treatment

3.6 Pre Exposure Prophylaxis (PrEP) Services

A. PrEP_NEW		MALE		FEMALES		TOTAL
15-19 Yrs	HIV-5185		HIV-5191		HIV-5196	
20-24 Yrs	HIV-5186		HIV-5192		HIV-5197	
25-49 Yrs	HIV-5187		HIV-5193		HIV-5198	
50+ Yrs	HIV-5188		HIV-5194		HIV-5199	
Totals	HIV-5189		HIV-5195		HIV-5200	

B. PrEP_CT		MALE		FEMALES		TOTAL
15-19 Yrs	HIV-5201		HIV-5206		HIV-5211	
20-24 Yrs	HIV-5202		HIV-5207		HIV-5212	
25-49 Yrs	HIV-5203		HIV-5208		HIV-5213	
50+ Yrs	HIV-5204		HIV-5209		HIV-5214	
Totals	HIV-5205		HIV-5210		HIV-5215	

C. PrEP_NEW: Dissagregated by Population Type				C. PrEP_CT: Dissagregated by Population Type		
PBFW	HIV-5216			PBFW	HIV-5222	
Adolescents	HIV-5217			Adolescents	HIV-5223	
Discordant Couples	HIV-5218			Discordant Couple:	HIV-5224	
Key Population	HIV-5219			Key Population	HIV-5225	
Others	HIV-5220			Others	HIV-5226	
Total PrEP	HIV-5221			Total PrEP	HIV-5227	

D. PrEP_CONT. (Consecutive 3 months)

		MALES		FEMALES		TOTAL
15-19 Yrs	HIV-5228		HIV-5233		HIV-5238	
20-24 Yrs	HIV-5229		HIV-5234		HIV-5239	
25-49 Yrs	HIV-5230		HIV-5235		HIV-5240	
50+ Yrs	HIV-5231		HIV-5236		HIV-5241	
Totals	HIV-5232		HIV-5237		HIV-5242	

E. PrEP_TOX (Adverse Events & Toxicity)

		**MALES**		**FEMALES**		**TOTAL**
15-19 Yrs	HIV-5243		HIV-5248		HIV-5253	
20-24 Yrs	HIV-5244		HIV-5249		HIV-5254	
25-49 Yrs	HIV-5245		HIV-5250		HIV-5255	
50+ Yrs	HIV-5246		HIV-5251		HIV-5256	
Total	HIV-5247		HIV-5252		HIV-5257	

F. PrEP_TOX (Adverse Events & Toxicity)

Death	HIV-5269	
Life threatening	HIV-5270	
Disability	HIV-5271	
Hospitalization	HIV-5272	
Congenial abnormality	HIV-5273	
Others	HIV-5274	

G. PrEP_DISC (Discontinued on PrEP)

		**MALES**		**FEMALES**		**TOTAL**
15-19 Yrs	HIV-5254		HIV-5259		HIV-5264	
20-24 Yrs	HIV-5255		HIV-5260		HIV-5265	
25-49 Yrs	HIV-5256		HIV-5261		HIV-5266	
50+ Yrs	HIV-5257		HIV-5262		HIV-5267	
Total	HIV-5258		HIV-5263		HIV-5268	

H. PrEP_DISC (Reasons)

Seroconversion to HIV	HIV-5269	
No longer at Risk	HIV-5270	
Poor Adherence	HIV-5271	
Contraindication to PrEP	HIV-5272	

APPENDIX 9: ADVERSE DRUG REACTION, MEDICATION ERROR, AND PRODUCT QUALITY REPORTING FORM

NPVF 001 version 00
November 2015

ADVERSE DRUG REACTION, MEDICATION ERROR AND PRODUCT QUALITY PROBLEM REPORTING FORM

(Identities of reporter and patient will remain strictly confidential)

NATIONAL PHARMACOVIGILANCE UNIT (NPVU)

The Director General
The Zambia Medicines Regulatory Authority
Plot No. 6903, Tuleteka Rd, Off Makishi Rd,
P.O. Box 31890, Lusaka, Zambia.

Telephone: +260211220429
Telefax: +260211238458
Email: pharmacy@zamra.co.zm

PATIENT INFORMATION

Patient initials: .... File No. .... Age: .... Weight (kg): ....

Sex: Male ☐ Female ☐ Date of birth: .... /.... /......... Height (cm): ....

DETAILS OF ADVERSE DRUG REACTION OR PRODUCT QUALITY PROBLEM

I am reporting on: 1) an Adverse Drug Reaction ☐ Date of onset of reaction: .... /....... /..............

2) a Product Quality Problem ☐ Category: medicine ☐ medical device ☐

Description of Adverse Drug Reaction or Product Quality Problem: ....

....

....

1. MEDICINES/ VACCINES/ MEDICAL DEVICES: *(✓) Tick against the* ***suspected*** *medicine/ vaccine*

Indicate all medicines the patient is taking

(✓)	Trade/ Generic Name & Batch Number	Dosage & dosing frequency	Route of administration	Start date (dd/mm/yy)	Stop date (dd/mm/yy)	Reasons for use

ADVERSE DRUG REACTION OUTCOME: ***(Tick all that apply)***

Outcome: ☐ Death ☐ Life threatening ☐ Disability ☐ Hospitalization ☐ Congenital abnormality

☐ Other *(specify)*: ....

Recovered: ☐ Yes ☐ No If YES, date of recovery: .... /....... /..............

Additional information *(e.g. Relevant medical history, medicines taken in the last 28 days, allergies, previous exposure, baseline test results/ lab data)*......... ....

....

2. PRODUCT QUALITY PROBLEM

Trade Name	Batch Number	Registration Number	Dosage Form & Strength	Expiry Date (mm/yyyy)	Size/ Type of container

Product sample(s) have been submitted for evaluation: ☐ Yes ☐ No Number of submitted samples: ☐

DETAILS OF REPORTER

Name: .... Profession: .... Signature: .... Date (dd/mm/yyyy):...........

Contact address: .... Phone: .... Email: ....

APPENDIX 10: PrEP PERFORMANCE INDICATOR REFERENCE TABLE FOR CORE INDICATORS.

INDICATOR	1. PrEP DEMAND CREATION ACTIVITIES AT COMMUNITY AND HEALTH FACILITY LEVEL - PrEP_REFER
**Indicator Definition**	PrEP_REFER: Number of HIV negative individuals referred for PrEP services within in the reporting period.
**Description**	This indicator records the numbers of HIV negative individuals referred from either community or health facility to be fully assessed for PrEP eligibility (i.e., both behaviourally and clinically)
**Numerator**	Number of individuals HIV negative individuals referred for PrEP services within the reporting period
**Denominator**	N/A
**Calculation**	Numerator
**Data Collection**	PrEP_REFER should be equal or greater than PrEP_SCREEN. As the demand creation activity increases for PrEP at both community and facilities, more HIV negative individuals should be screened for substantial risk of HIV acquisition and referred for full PrEP screening services by community workers and lay counsellors at HTS SDPs. At facility level, HTS Register will be used to document the numbers of PBFW, Sero-discordant couples, AGYW and other population with indication of substantial risks of HIV while the community system will track same with HIV Prevention Outreach Register in use.
**Frequency**	Data should be collected continuously at the community and facility level, aggregated periodically, and aligned with the reporting frequency of other routinely collected indicators (monthly, quarterly, semi-annual or as recommended). These data should then be combined for annual reporting.
**Disaggregation**	• Age (16–19, 20–24, 25–49, and 50+ years)<br>• Sex (male, female)<br>• Sub-population types: (PBFW, adolescents, discordant couples, key populations (men who have sex with men, sex workers, people who inject drugs, people in prisons and other closed settings, and transgender), and others<br>• Province, district, facility, and community.

INDICATOR	2. PrEP SCREENING COVERAGE- PrEP_SCREEN_COV
**Indicator Definition**	PrEP_SCREEN_COV: Percentage of HIV negative individuals screened for PrEP services of the target group (estimated).
**Description**	This indicator measures and records the proportion of the estimated HIV negative individuals within the target groups assessed for PrEP eligibility within facility and community-based program (both behaviourally and clinically)
**Numerator**	Number of HIV-negative individuals screened for PrEP services of the target group within the reporting period (i.e., PrEP_SCREEN)
**Denominator**	Estimated total number of HIV negative individuals due for screening within the same target group.
**Calculation**	Number screened for PrEP eligibility (PrEP_SCREEN) in the target group/ Estimated total number of HIV negative individuals due for screening within the same target group *100
**Data Collection**	PrEP_SCREEN_COV per target group for should be less than or equal to 100%. As the demand increases for PrEP, more individuals should be screened for PrEP services at both the facility and community program level. Each target group PrEP_SCREEN_COV should be increasing corresponding to the sum efforts of all the PrEP IPs in the country. The Denominator for this indicator will be determined at national level based on statistical projections and size estimation or a combination of both.
**Frequency**	Numerator for this data should be collected continuously at the facility and community level, aggregated periodically, and aligned with the reporting frequency of other routinely collected indicators (monthly, quarterly, semi-annual, or as recommended) at national and sub-national level. The established Denominator can be applied at national and sub-national level to provide feedbacks on the target group lagging. These data should then be combined for annual reporting.
**Disaggregation**	• Age (16–19, 20–24, 25–49, and 50+ years)<br>• Sex (male, female)<br>• Sub-population types: (PBFW, adolescents, discordant couples, key populations (men who have sex with men, sex workers, people who inject drugs, people in prisons and other closed settings, and transgender), and others<br>• Province, district, facility, and community.

INDICATOR	3. PrEP SCREENING - PrEP_SCREEN
**Indicator Definition**	PrEP_SCREEN: Number of HIV negative individuals screened for PrEP services within in the reporting period.
**Description**	This indicator records the numbers of HIV negative individuals screened for PrEP eligibility i.e., full clinical and non-clinical eligibility through both community and facility-based PrEP service delivery points.
**Numerator**	Number of HIV-negative individuals screened for PrEP services within the reporting period
**Denominator**	N/A
**Calculation**	Numerator
**Data Collection**	PrEP_SCREEN should be equal or greater than PrEP_ELIGIB. As the demand increases for PrEP, more individuals should be screened for PrEP services at both the facility and community program level.
**Frequency**	Data should be collected continuously at the facility level, aggregated periodically, and aligned with the reporting frequency of other routinely collected indicators (monthly, quarterly, semi-annual, or as recommended). These data should then be combined for annual reporting.
**Disaggregation**	• Age (16–19, 20–24, 25–49, and 50+ years)<br>• Sex (male, female)<br>• Sub-population types: (PBFW, adolescents, discordant couples, key populations (men who have sex with men, sex workers, people who inject drugs, people in prisons and other closed settings, and transgender), and others<br>• Province, district, facility, and community.

INDICATOR	4. PrEP ELIGIBILITY – PrEP_ELIGB
**Indicator Definition**	PrEP_ELIGB: Percentage of screened individuals who are eligible for PrEP services within the reporting period.
**Description**	This indicator records the percentage of all individuals screened who are eligible for PrEP services. This data is then compared to the PrEP_UP to understand what percentage of eligible individuals are initiated on PrEP. Eligibility should include at a minimum: 1) HIV-negative status and 2) no signs and symptoms of acute HIV. The third criteria, whether an individual is at substantial risk for HIV and may benefit from PrEP, will be contextual and should be based on national guidelines. The main factors that influence HIV risk are a person's own and their partner(s)' sexual and drug-using behavior, their partner(s)' HIV status, the overall background HIV prevalence and incidence where they live and the sub-population to which they may belong. Populations to whom PrEP may be offered as a priority may include key populations (men who have sex with men, sex workers, people who inject drugs, people in prisons and other closed settings, and transgender), serodiscordant couples, and other priority populations such as adolescent girls and young women in certain high incidence settings.<br><br>Contraindications for PrEP include HIV infection, signs/symptoms of acute HIV infection among people who have had a recent HIV exposure, probable recent exposure to HIV, estimated creatinine clearance of less than 60ml/min (if known) and allergy or contraindication to any medicine in the PrEP regimen. More details are provided in the national clinical guidelines.
**Numerator**	Number of individuals screened and eligible for PrEP services within the reporting period
**Denominator**	Number of individuals screened for PrEP services within the reporting period (PrEP_SCREEN)
**Calculation**	Numerator/denominator*100
**Data Collection Methodology**	Ideally, all the PrEP_ELIGB numerator clients are expected to initiate PrEP within the reporting period when they are eligible for PrEP. Hence, PrEP_ELIGB numerator should be equal or greater than PrEP_NEW for the same reporting period unless there are documentation issues or errors in the data.<br><br>On no account will PrEP_ELIGB numerator be greater than PrEP_SCREEN for any site. This can be used as one of the data quality checks. Data inconsistencies will trigger data quality reviews to improve of the M&E system.
**Frequency**	Data should be collected continuously at the facility level, aggregated periodically, and aligned with the reporting frequency of other routinely collected indicators (monthly, quarterly, semi-annual, or as recommended). These data should then be combined for annual reporting.
**Disaggregation**	• Age (16–19, 20–24, 25–49, and 50+ years)<br>• Sex (male, female)<br>• Sub-population types: (PBFW, adolescents, discordant couples, key populations (men who have sex with men, sex workers, people who inject drugs, people in prisons and other closed settings, and transgender), and others<br>• Province, district, facility, and community.

INDICATOR	5. PrEP UPTAKE* - PrEP_UP
**Indicator Definition**	PrEP_UP: Percentage of eligible people who initiated oral antiretroviral PrEP within the reporting period
**Description**	This indicator is key to assessing uptake of PrEP among those who are eligible. The numerator of this indicator is aligned with the PEPFAR MER guidance (Version 2.6), people who initiated oral PrEP includes only those who started PrEP for the first time (excluding those who have discontinued PrEP and restarted PrEP) within the reporting period.<br>Patients counted here as eligible for starting PrEP will be in line with the Zambia national consolidated guidelines for PrEP.
**Numerator**	PrEP_NEW: Number of individuals who were newly enrolled on PrEP to prevent HIV infection in the reporting period.
**Denominator**	PrEP_ELIGIB (Numerator): Number of individuals screened who were eligible for PrEP services in the reporting period.
**Calculation**	Numerator/Denominator*100
**Data Collection Methodology**	The numerator is generated by counting the number of people who were newly initiated oral PrEP during the reporting period, among those who were screened and eligible for PrEP within the same reporting period. The numerator only includes people who received PrEP for the first time, those who had previously discontinued PrEP and restarted PrEP in the reporting period are excluded. This definition is aligned with MER guidance (V2.6).[46] Regular PrEP users who are continuing PrEP should be excluded from both the numerator and denominator. Individuals who were classified as "Used PrEP before" in the Prevention section of the Initial PrEP Form (Appendix 1). The numerator should count everyone only once in each reporting period. All people who received oral PrEP through national programs, demonstration projects, research or through private means and are taking it according to WHO/Zambia standards should be included. An individual should only be counted once in each reporting period even if they initiated PrEP more than once after a period of discontinuation. Age is defined as the age at the time the person initiates PrEP.
**Frequency**	Data should be collected continuously at the facility level, aggregated periodically, and aligned with the reporting frequency of other routinely collected indicators (monthly, quarterly, semi-annual, or as recommended). These data should then be combined for annual reporting.
**Disaggregation**	• People who received PrEP for the first time in their lives<br>• Age (16–19, 20–24, 25–49, and 50+ years)<br>• Sex (male or female)<br>• Sub-population types: (PBFW, adolescents, discordant couples, key populations (men who have sex with men, sex workers, people who inject drugs, people in prisons and other closed settings, and transgender), and others<br>• Province, district, facility, and community.

INDICATOR	6. EARLY CONTINUATION ON PrEP* - PrEP_EARLY
**Indicator Definition**	PrEP_EARLY: Percentage of PrEP users who continued on oral PrEP for three consecutive months after having initiated PrEP within the reporting period
**Description**	This indicator measures the continuation of PrEP among people who started on PrEP and also assesses early loss to follow-up. Early experience in demonstration projects and small programs indicates that many users who discontinue oral PrEP do so during the first few months. This indicator provides a measure of early PrEP discontinuation as well as an indication of the number likely to continue taking PrEP. Furthermore, risk for HIV (and, therefore, need for oral PrEP use) is unlikely to change in a period shorter than three months, although it is possible.<br><br>If the percentage of people who continue on PrEP at three months is low, further investigation into the reasons that people stopped taking PrEP (whether due to side-effects, changes in behavior/risk or structural factors) could be determined and programs adjusted as needed.
**Numerator**	Number of people who continued on PrEP for three consecutive months after having initiated PrEP in the reporting period.
**Denominator**	Number of individuals who were initiated PrEP (PrEP_NEW) within the reporting period.
**Calculation**	Numerator/Denominator*100
**Data Collection Methodology**	The numerator is generated by counting the number of people who initiated oral PrEP in the reporting period and who continued PrEP for three consecutive months as a cohort.<br><br>Some PrEP visit schedules may include a first visit one month after PrEP initiation, a second visit two months later, and visits every three months thereafter. In this case, the continuation indicator would be measured at the second visit, three months after PrEP initiation. This measurement periodicity will be consistent with programs that only have three monthly visits and will also comply with routine quarterly reporting. All people who return for the three-month visit and took PrEP until that time should be counted, whether they chose to continue with PrEP after the three-month visit.<br><br>An individual should only be counted once in a given reporting period even if they were offered PrEP more than once, as may happen if someone initiates, discontinues, and restarts PrEP in the same reporting period. Individuals who initiate PrEP in the last 2 months of the reporting period will have their continuation appointments in the next reporting period. These individuals should be counted in the period in which they initiated PrEP. Age is defined as the age at the time the person initiates PrEP.
**Frequency**	Data should be collected continuously at the facility level, aggregated periodically, and aligned with the reporting frequency of other routinely collected indicators (monthly, quarterly, semi-annual or as recommended). These data should then be combined for annual reporting. These data should be organized as a cohort report for a three months follow-up of people who initiated PrEP at the same time.
**Disaggregation**	• Age (16–19, 20–24, 25–49, and 50+ years)<br>• Sex (male, female)<br>• Sub-population types: (PBFW, adolescents, discordant couples, key populations (men who have sex with men, sex workers, people who inject drugs, people in prisons and other closed settings), and others<br>• Province, district, facility, and community.

INDICATOR	7. PrEP ASSOCIATED TOXICITY PREVALENCE* - PrEP_TOX
**Indicator Definition**	PrEP_TOX: Percentage of people who received oral PrEP who have discontinued or interrupted PrEP due to a serious ARV-associated toxicity within three months.
**Description**	Toxicity prevalence associated with PrEP is expected to be low. However, experience with large-scale PrEP programs and longer exposure has been limited. Therefore, active surveillance and toxicity monitoring for people using PrEP is important in order to identify potential adverse outcomes that may arise as PrEP programs scale up and reach larger numbers of people.<br><br>The major expected toxicities related to the use of PrEP are bone and renal toxicities associated with tenofovir in populations with associated risk factors. During pregnancy, there is growing evidence for the safety of tenofovir and/or emtricitabine when used for treatment and prevention of mother-to-child transmission in HIV- or HBV-infected pregnant women. Additional surveillance programs are needed to evaluate PrEP safety during pregnancy and breastfeeding (see section below on special considerations during pregnancy). The 2016 WHO Consolidated ARV Guidelines state that there is no safety-related rationale for disallowing or discontinuing PrEP use during pregnancy and breastfeeding for HIV-negative women who remain at risk of HIV acquisition.<br><br>Adverse drug reactions leading to PrEP discontinuation or interruption should be routinely recorded in an appropriate PrEP register.
**Numerator**	Number of people who received oral PrEP and have discontinued or interrupted PrEP due to a serious ARV related toxicity within the reporting period .
**Denominator**	Number of people who received oral PrEP at least once within the reporting period.
**Calculation**	Numerator/Denominator*100
**Data Collection Methodology**	The numerator is generated by counting the number of people taking oral PrEP who have discontinued or interrupted PrEP due to PrEP-limiting serious adverse drug toxicity within the reporting period , defined as a life-threatening illness, death, hospitalization or disability or any adverse drug reaction that resulted in PrEP discontinuation.<br><br>The denominator is generated by counting the number of people who received oral PrEP at least once during the reporting period in accordance with national guidelines. People who received oral PrEP at least once includes those who initiated PrEP for the first time and those who may have discontinued PrEP and restarted PrEP in the reporting period, as well as those who are continuing on PrEP. The denominator should include all people who received oral PrEP at least once during the reporting period.
**Frequency**	Data should be collected continuously at the facility level, aggregated periodically, and aligned with the reporting frequency of other routinely collected indicators (monthly, quarterly, semi-annual, or as recommended). These data should then be combined for annual reporting.
**Disaggregation**	• Age (16–19, 20–24, 25–49, and 50+ years)<br>• Sex (male, female)<br>• Sub-population types: (PBFW, adolescents, discordant couples, key populations (men who have sex with men, sex workers, people who inject drugs, people in prisons and other closed settings, and transgender), and others<br>• Province, district, and facility<br>• Type of adverse outcomes (death, life threatening, disability, hospitalization, congenital abnormality) as included in the adverse Drug Reaction, Medication Error and Product Quality Reporting Form (Appendix 9).

INDICATOR	8. HIV POSITIVITY AMONG PEOPLE WHO HAVE BEEN PRESCRIBED PrEP* - PrEP_HIVPOS
**Indicator Definition**	PrEP_HIVPOS: Percentage of people who test HIV-positive among people who received PrEP at least once in the reporting period and had at least one follow-up HIV test.
**Description**	This indicator measures the percentage of people who test HIV positive after being prescribed PrEP. HIV testing is required before starting PrEP, and regularly thereafter while taking PrEP. The HIV test to determine PrEP eligibility is not included in either the numerator or the denominator. The last recorded HIV test in the reporting period is counted. Using quality-assured HIV testing is important, and timely linkage of people who test positive to HIV treatment services is essential. PrEP is highly effective when taken as prescribed. People who test HIV positive after having received PrEP may have had acute undetected HIV infection when they started PrEP. Other potential reasons for testing positive are poor adherence which results in reduced concentrations of PrEP and reduced effectiveness in case of exposure, or the acquisition of drug-resistant virus.<br><br>Measuring the proportion of PrEP users who test HIV-positive is problematic in the absence of individual-level, longitudinal monitoring data. Determining the denominator of "all PrEP users" is hampered by differential follow-up and the unknown HIV status of those who are lost to follow-up. Furthermore, follow-up may differ by site, location or by population (for example, men versus women). Therefore, using a denominator of all people prescribed PrEP in a given period can be misleading, as apparent very low levels of HIV positivity could result equally from high adherence to PrEP or from non-adherence and loss to follow-up. Therefore, this indicator should be interpreted with caution, particularly in instances with high loss to follow up and may not be appropriate for comparisons across different service delivery types or locations.<br><br>This indicator is not a measure of PrEP efficacy. It serves as an indicator for program performance to further investigate the potential reasons for seroconversion, and if appropriate, to adjust programs (such as for eligibility screening or adherence counseling) as needed. It is difficult to accurately determine the reasons for seroconversion, or to assess adherence retrospectively. In order to minimize recall bias, asking PrEP users about recent adherence (over the past week or month) may be beneficial.
**Numerator**	Number of people who had a positive HIV follow-up test among people who received oral PrEP at least once within the reporting period.
**Denominator**	Number of people who received oral PrEP at least once within the reporting period, and who had at least one follow up HIV test.
**Calculation**	Numerator/denominator
**Data Collection Methodology**	The numerator is generated by counting the total number of people who have an HIV-positive follow-up test result among people who received oral PrEP at least once in the reporting period and who had at least one follow up HIV test. The numerator should not include people who last used PrEP greater than 6 months prior to the HIV test date.<br><br>The denominator is generated by counting the number of people who received PrEP at least once in the reporting period and who had at least one follow up HIV test taken in the reporting period. Only the most recent test result should be counted. For example, if a person used PrEP for 6 months, and during that time received 2 HIV-negative tests to enable them to continue on PrEP, their contribution to the denominator would be 1. Similarly, if a PrEP user only has 2 recorded HIV tests in the reporting period, one of which was HIV-positive, they would contribute 1 to the numerator and 1 to the denominator.<br><br>The first HIV test conducted to determine PrEP eligibility should not be included in the numerator or denominator. This test should be HIV-negative for all people who are prescribed PrEP. People who test HIV positive as part of PrEP eligibility screening should not be included in the numerator or denominator as they would not be prescribed PrEP.
**Frequency**	Data should be collected continuously at the facility level, aggregated periodically, and aligned with the reporting frequency of other routinely collected indicators (semi-annual or as recommended). These data should then be combined for annual reporting.
**Disaggregation**	• Age (16–19, 20–24, 25–49, and 50+ years)<br>• Sex (male, female)<br>• Sub-population types: (PBFW, adolescents, discordant couples, key populations (men who have sex with men, sex workers, people who inject drugs, people in prisons and other closed settings), and others<br>• Province, district, facility, and community.

INDICATOR	9. MER INDICATOR – INDIVIDUALS NEW ON PrEP † - PrEP_NEW
**Indicator Definition**	PrEP_NEW: Number of individuals who were enrolled on oral antiretroviral pre-exposure prophylaxis (PrEP) to prevent HIV infection in the reporting period.
**Description, Numerator, Denominator, Calculation, Data Collection, Frequency, and Disaggregation**	Please refer to the latest PEPFAR MER Indicator Reference Guide at https://www.state.gov/wp-content/uploads/2021/09/FY22-MER-2.6-Indicator-Reference-Guide.pdf

INDICATOR	10. MER INDICATOR – INDIVIDUALS ON PrEP † - PrEP_CT
**Indicator Definition**	PrEP_CT: Number of individuals that returned for a follow-up or re-initiation visit to receive PrEP during the reporting period.
**Description, Numerator, Denominator, Calculation, Data Collection, Frequency, and Disaggregation**	Please refer to the latest PEPFAR MER Indicator Reference Guide at https://www.state.gov/wp-content/uploads/2021/09/FY22-MER-2.6-Indicator-Reference-Guide.pdf

Indicator	11. PrEP continuation – PrEP_CONT
**Indicator Definition**	PrEP_CONT: Percentage of individuals active on PrEP at specific time period 6, 9, 12 and >12 months post initiation.
**Description**	This indicator is a proportion of PrEP clients who continued on PrEP within their respective cohorts of 6,9,12 and greater than 12 months follow up period. The indicator is very important to understanding cohort retention on PrEP and therefore may be useful in improving the clinical management, cohort tracking and monitoring of the PrEP clients at each site level.
**Numerator**	Number of individuals who have continued on PrEP continuously and attended their clinical appointment at the given period interval (6, 9, 12, >12 months)
**Denominator**	Number of individuals who were initiated in their respective cohort
**Calculation**	Numerator/denominator
**Data Collection Methodology**	This indicator is generated report of a cohort follow-up of clients on PrEP at 6,9, 12 and >12 months. It's therefore proportion of clients who initiated on PrEP as the same cohort/period and when follow-up to ascertain their status on PrEP at a specific point in time, they were still receiving PrEP in a continuation phase. In accordance with the national PrEP Implementation guidelines, PrEP clients are expected to return every 3 months for follow on visits. These visits and the clinical encounters are captured on PrEP Follow-up Form or SmartCare and summarized into the National PEP/PrEP Registers for collation in sites and other client-level data platforms such as DHIS2 Tracker.
**Frequency**	Data should be collected continuously at the facility level, aggregated periodically, and aligned with the reporting frequency of other routinely collected indicators (semi-annual or as recommended). These data should then be combined for annual reporting.
**Disaggregation**	• Age (16–19, 20–24, 25–49, and 50+ years)<br>• Sex (male, female)<br>• Sub-population types: (PBFW, adolescents, discordant couples, key populations (men who have sex with men, sex workers, people who inject drugs, people in prisons and other closed settings, and transgender), and others<br>• Province, district, facility, and community

INDICATOR	12. PrEP DISCONTINUATION AND REASONS – PrEP_DISC
**Indicator Definition**	PrEP_DISC: Percentage of individuals discontinued on PrEP in the reporting period.
**Description**	This indicator attempts to help in the tracking and documentation of the number of individuals discontinuing from PrEP at a specific time and most especially the specific reason attributed to the discontinuation. Understanding the key reasons will enable for a better and more efficient PrEP programming.
**Numerator**	Number of individuals who discontinued on PrEP in the reporting period
**Denominator**	Number of individuals who were initiated in the reporting period.
**Calculation**	Numerator/denominator
**Data Collection Methodology**	This indicator is generated report of a cohort follow-up of clients on PrEP. Therefore, it is the number of clients who discontinued on PrEP at any specific time period and the reasons for the discontinuation. The reasons are included in the PrEP Follow Up Form under the Plan section (reasons for stopping include sero conversion to HIV, no longer at risk, poor adherence, and contraindication to PrEP).<br><br>In accordance with the national PrEP Implementation guidelines, PrEP clients are expected to return for 3, 6, 12 months and other follow-on visits, these visits and the clinical encounters are usually to be captured on PrEP Follow-up Form is the summarized into the National PEP/PrEP Registers for collation in sites without EMR such as SmartCare or other client-level data platform such as DHIS2 Tracker.
**Frequency**	Data should be collected continuously at the facility level, aggregated periodically, and aligned with the reporting frequency of other routinely collected indicators (semi-annual or as recommended). These data should then be combined for annual reporting.
**Disaggregation**	• Age (16–19, 20–24, 25–49, and 50+ years)<br>• Sex (male, female)<br>• Sub-population types: (PBFW, adolescents, discordant couples, key populations (men who have sex with men, sex workers, people who inject drugs, people in prisons and other closed settings, and transgender), and others<br>• Province, district, facility, and community<br>• Reasons for stopping include seroconversion to HIV, no longer at risk, poor adherence, and contraindication to PrEP.

* Indicators were adapted from the WHO Implementation tool for PrEP of HIV Infection. [27]

† Indicators from the PEPFAR Monitoring, Evaluation and Reporting Indicator Reference Guide. [46]

APPENDIX 11: NATIONAL PrEP PROGRAM INDICATOR MATRIX

Indicator Code<br><br>Indicator Type	Description	Indicator Calculation	Indicator Disaggregation	Data Required to Calculate	Data Source	Frequency	Indicator Targets Year 1	Indicator Target Year 2	Indicator Target Year 3	Responsible	Purpose (How will the data be used)
**PrEP_REFER**<br><br>Process, Count	*Number of HIV-Negative individuals referred for PrEP screening.*<br>This indicator records the numbers of HIV negative individuals referred from either community or health facility to be fully assessed for PrEP eligibility (both behaviourally and clinically)	No calculation required. A count of all HIV negative individuals screened for PrEP eligibility during HIV Prevention intervention session	**Sub-population types:** (PBFW, adolescents, discordant couples, key populations (men who have sex with men, sex workers, people who inject drugs, people in prisons and other closed settings, and transgender), and others Province, district, and community.	Assessment for substantially risk of HIV acquisition at the community and facility-based service delivery points (SDPs)	PrEP non-clinical form<br>HIV prevention outreach register<br>HIV Counselling and Testing Register	Monthly, Quarterly, Annually	TBD	TBD	TBD	PrEP IPs, PHOs, DHOs and MOH	As the demand creation activities for PrEP increases at both the community and health facility, more HIV negative individuals within each target groups should be getting referrals for full screening for PrEP eligibility.
**PrEP_SCREEN**<br><br>Process, Count	*Number of HIV negative individuals screened for PrEP services within in the reporting period.*<br>This indicator records the numbers of individuals screened for PrEP eligibility (both behaviourally and clinically)	A count of all HIV negative individuals screened for PrEP full clinical and non-clinical eligibility through both community and facility-based PrEP service delivery points.	**Sub-population types:** PBFW, adolescents, discordant couples, key populations (men who have sex with men, sex workers, people who inject drugs, people in prisons and other closed settings, and transgender), and others. Province, district, facility, and community.	PrEP eligibility assessment at the facility and community-based PrEP program	PrEP initial form<br>SmartCare	Monthly, Quarterly, Annually	TBD	TBD	TBD	PrEP IPs, Facilities, PHOs, DHOs and MOH	As the demand creation activities for PrEP increases at both the health facilities and community, there should be corresponding increase in numbers of individuals screened for PrEP services
**PrEP_SCREEN _COV**<br><br>Process/ Coverage, Percentage	*Percentage of HIV negative individuals screened for PrEP services of the target group (estimated).*<br>This indicator measures and records the proportion of the estimated HIV negative individuals within the target groups assessed for PrEP eligibility within facility and community-based program (both behaviourally and clinically)	Number screened for PrEP eligibility (PrEP_SCREEN) in the target group/ Estimated total number of HIV negative individuals due for screening within the same target group *100	**Sub-population types:** PBFW, adolescents, discordant couples, key populations (men who have sex with men, sex workers, people who inject drugs, people in prisons and other closed settings, and transgender), and others Province, district, and community.	PrEP eligibility assessment at the community-based PrEP program	HIV prevention outreach register<br>PrEP non-clinical form<br>Estimates for each Target Group	Quarterly, Annually	TBD	TBD	TBD	PrEP IPs, PHOs, DHOs and MOH	As the demand creation activities for PrEP increases, higher proportion of HIV negative individuals within each target groups should have been assessed/screened for eligibility for PrEP services.

Indicator Code<br><br>Indicator Type	Description	Indicator Calculation	Indicator Disaggregation	Data Required to Calculate	Data Source	Frequency	Indicator Targets Year 1	Indicator Target Year 2	Indicator Target Year 3	Responsible	Purpose (How will the data be used)
**PrEP_ELIGIB**<br><br>Output/ Coverage, Percentage	*Percentage of screened individuals who are eligible for PrEP services.*<br>This indicator records the percentage of all individuals screened who are eligible for PrEP services.	Number eligible for PrEP/ PrEP_SCREEN*100	**Sub-population types:** PBFW, adolescents, discordant couples, key populations (men who have sex with men, sex workers, people who inject drugs, people in prisons and other closed settings, and transgender), and others.<br>Province, district, facility, and community.	PrEP eligibility assessment at the facility and community-based PrEP program	PrEP initial form SmartCare	Monthly, Quarterly, Annually	TBD	TBD	TBD	PrEP IPs, Facilities, PHOs, DHOs and MOH	This data - PrEP_ELIGIB will help IPs and Facility Managers to understand and predict what percentage of eligible individuals (per target group) would need PrEP services and thereby may help with commodity forecasts
**PrEP_UP**<br><br>Output/Service Coverage, Percentage	*Percentage of eligible people who were initiated on antiretroviral PrEP.*<br>This indicator records the numbers of individuals who adopted PrEP for the first time amongst those considered PrEP eligible. This indicator is key to assessing uptake of PrEP among those who are eligible.	PrEP_NEW/ PrEP_ELIGB (Numerator)*100	**Age** (16–19, 20–24, 25–49, and 50+)<br>**Sex**<br>**Sub-population types:** PBFW, adolescents, discordant couples, key populations (men who have sex with men, sex workers, people who inject drugs, people in prisons and other closed settings, and transgender), and others Province, district, facility, and community.	PrEP service delivery points at the facility and community-based PrEP program	PrEP initial form SmartCare	Monthly, Quarterly, Semi-annually, Annually	TBD	TBD	TBD	PrEP IPs, Facilities, PHOs, DHOs and MOH	This data - PrEP_UP will help IPS and Facility Managers understand and predict by age category, Sex and Sub-population types dropping off PrEP initiation and then plan for enquiry and suitable intervention.
**PrEP_NEW**<br><br>Output, Count	*Number of individuals who were newly enrolled on antiretroviral PrEP within in the reporting period.*<br>This indicator records the numbers of ARV naïve HIV negative individuals eligible for PrEP and adopting it as HIV prevention technology for the first time ever.	A count of all ARV naïve HIV negative individuals adopting PrEP for the first time.	**Age** (16–19, 20–24, 25–49, and 50+)<br>**Sex** (male or female)<br>**Sub-population types:** (PBFW, adolescents, discordant couples, key populations (men who have sex with men, sex workers, people who inject drugs, people in prisons and other closed settings, and transgender), and others Province, district, facility, and community.	PrEP service delivery points at the facility and community-based PrEP program	PrEP initial form PrEP register SmartCare	Monthly, Quarterly, Semi-annually, Annually	TBD	TBD	TBD	PrEP IPs, Facilities, PHOs, DHOs and MOH	In response to increased demand creation and screening for PrEP amongst target groups, result for this indicator ought to be in proportion to other indicators such as PrEP_ELIGIB and PrEP_UP provided there are no stock-outs of commodities.

Indicator Code<br><br>Indicator Type	Description	Indicator Calculation	Indicator Disaggregation	Data Required to Calculate	Data Source	Frequency	Indicator Targets Year 1	Indicator Target Year 2	Indicator Target Year 3	Responsible	Purpose (How will the data be used)
**PrEP_CT**<br><br>Outcome, Count	*Number of individuals that returned for a follow-up or re-initiation visit to receive PrEP during the reporting period*<br>This indicator records the numbers of individuals HIV negative on follow-up PrEP clinical visits/Refill appointment in addition to prior PrEP clients restarting PrEP after a period of stopping PrEP	A count of all PrEP clients on follow-up clinical visits or ARV refill in addition to prior PrEP clients restarting PrEP after a period of stopping PrEP.	**Age** (16–19, 20–24, 25–49, and 50+)<br>**Sex**<br>**Sub-population types:** (PBFW, adolescents, discordant couples, key populations (men who have sex with men, sex workers, people who inject drugs, people in prisons and other closed settings, and transgender), and others<br>Province, district, facility, and community.	PrEP service delivery points at the facility and community-based PrEP program	PrEP Follow-up form<br>PrEP register<br>SmartCare	Monthly, Quarterly, Semi-annually Annually	TBD	TBD	TBD	PrEP IPs, Facilities, PHOs, DHOs and MOH	This indicator helps program managers to understand issues around continuity on PrEP services for each target group and to take appropriate steps to initiative follow-up tracking of missed appointments.
**PrEP_EARLY**<br><br>Outcome, Percentage	*Percentage of individuals who continued on PrEP for three consecutive months post initiation*<br>This indicator measures the continuation of PrEP among people who started on PrEP and also assesses early loss to follow-up.	Number of individuals on PrEP for 3 consecutive months post-initiation/PrEP_NEW cohort for the same period *100	**Age** (16–19, 20–24, 25–49, and 50+)<br>**Sex**<br>**Sub-population types:** PBFW, adolescents, discordant couples, key populations (men who have sex with men, sex workers, people who inject drugs, people in prisons and other closed settings, and transgender), and others<br>Province, district, facility, and community.	PrEP service delivery points at the facility and community-based PrEP program	PrEP initial form<br>PrEP Follow-up form<br>PrEP Register<br>SmartCare	Quarterly and Annually	TBD	TBD	TBD	PrEP IPs, Facilities, PHOs, DHOs and MOH	Help measure early adherence and early loss-to-follow-up on PrEP services program.

Indicator Code<br><br>Indicator Type	Description	Indicator Calculation	Indicator Disaggregation	Data Required to Calculate	Data Source	Frequency	Indicator Targets Year 1	Indicator Target Year 2	Indicator Target Year 3	Responsible	Purpose (How will the data be used)
**PrEP_TOX**<br><br>Patient Safety, Percentage	*Percentage of people who received PrEP who have discontinued or interrupted PrEP within a three-month period due to a serious ARV-associated toxicity.*<br>This indicator records the numbers of individuals using PrEP who have discontinued or interrupted PrEP due to PrEP-limiting serious adverse drug toxicity within the reporting period, defined as a life-threatening illness, death, hospitalization or disability or any adverse drug reaction that resulted in PrEP discontinuation	Number of individuals who discontinued or interrupted PrEP within 3 months post-initiation/PrEP_NEW cohort for the same period *100	**Age** (16–19, 20–24, 25–49, and 50+ years)<br>**Sex**<br>**Sub-population types:** PBFW, adolescents, discordant couples, key populations (men who have sex with men, sex workers, people who inject drugs, people in prisons and other closed settings, and transgender), and others Province, district, facility, and community.	PrEP service delivery points at the facility and community-based PrEP program	Adverse drug reaction, medication error, and product quality reporting form SmartCare	Quarterly, Annually	TBD	TBD	TBD	PrEP IPs, Facilities, PHOs, DHOs and MOH	The indicator is to help in active surveillance and toxicity monitoring amongst people using PrEP to identify potential adverse outcomes arising from scale up of PrEP services.
**PrEP_HIVPOS**<br><br>Outcome	*Percentage of people who test HIV-positive among people who received PrEP at least once in the reporting period and had at least one follow-up HIV test within the reporting period.*<br>This indicator measures the percentage of people who test HIV positive after being prescribed PrEP.	Number of individuals who tested HIV positive post-initiation on PrEP/PrEP_CT with follow-up HIV test *100	**Age** (16–19, 20–24, 25–49, and 50+)<br>**Sex** (male or female)<br>**Sub-population types:** PBFW, adolescents, discordant couples, key populations (men who have sex with men, sex workers, people who inject drugs, people in prisons and other closed settings, and transgender), and others Province, district, facility, and community.	PrEP service delivery points at the facility and community-based PrEP program	PrEP initial form<br>PrEP Follow-up form<br>SmartCare	Quarterly, Annually	TBD	TBD	TBD	PrEP IPs, Facilities, PHOs, DHOs and MOH	This indicator is designed to further investigate the potential reasons for seroconversion, and to adjust programs (such as for eligibility screening or adherence counseling) as needed.

Indicator Code<br><br>Indicator Type	Description	Indicator Calculation	Indicator Disaggregation	Data Required to Calculate	Data Source	Frequency	Indicator Targets Year 1	Indicator Target Year 2	Indicator Target Year 3	Responsible	Purpose (How will the data be used)
**PrEP_CONT**<br><br>Outcome, Percentage	*Percentage of individuals active on PrEP (6, 9, 12 and >12 months).*<br>This indicator is a proportion of PrEP clients who continued on PrEP within their respective cohorts of 6,9,12 and greater than 12 months follow up period.	Number of individuals active on PrEP at 6,9,12 and >12months post-initiation/Number of individuals who were initiated in their respective cohort at 6,9,12 and >12months * 100	**Age** (16–19, 20–24, 25–49, and 50+)<br>**Sex**<br>**Sub-population types:** PBFW, adolescents, discordant couples, key populations (men who have sex with men, sex workers, people who inject drugs, people in prisons and other closed settings, and transgender), and others<br>Province, district, facility, and community.	PrEP service delivery points at the facility and community-based PrEP program	PrEP follow up form SmartCare	Quarterly, Annually	TBD	TBD	TBD	PrEP IPs, Facilities, PHOs, DHOs and MOH	The indicator is very important to understanding cohort retention on PrEP and therefore may be useful in improving the clinical management, cohort tracking and monitoring of the PrEP clients at each site level.
**PrEP_DISC**<br><br>Outcome, Percentage	*Percentage of individuals discontinued on PrEP.*<br>This indicator attempts to help in the tracking and documentation of the number of individuals discontinuing from PrEP at a specific time and most especially the specific reason attributed to the discontinuation.	Number of individuals who discontinued on PrEP in the reporting period/Number of individuals who were initiated in the reporting period * 100<br><br>The reason for discontinuation must be stated for each individual	**Age** (16–19, 20–24, 25–49, and 50+)<br>**Sex**<br>**Sub-population types:** PBFW, adolescents, discordant couples, key populations (men who have sex with men, sex workers, people who inject drugs, people in prisons and other closed settings, and transgender), and others<br>Province, district, facility, and community.	PrEP service delivery points at the facility and community-based PrEP program	PrEP follow up form SmartCare	Quarterly, Annually	TBD	TBD	TBD	PrEP IPs, Facilities, PHOs, DHOs and MOH	This indicator will help program managers understand the key reasons for PrEP discontinuation and will enable for a better and more efficient PrEP programming.

Appendix 12: Mapping of the Indicators to the PrEP Cascade

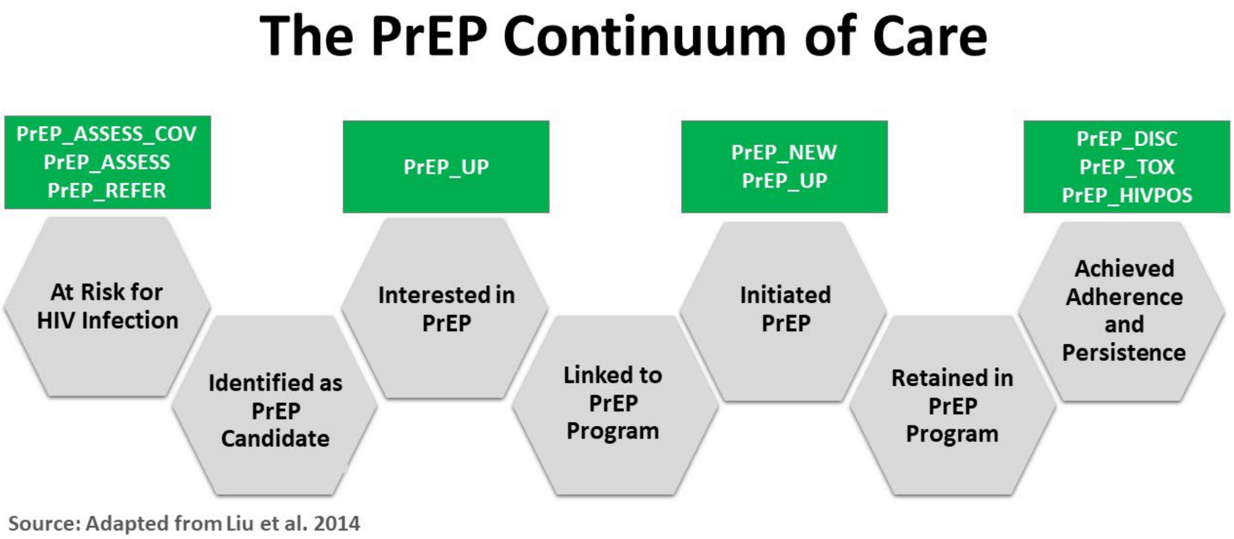

REFERENCES

1. Ministry of Health Zambia. Zambia Population-based HIV Impact Assessment (ZAMPHIA) 2016: Final Report. In. Lusaka, Zambia: Ministry of Health; 2016.
2. Zambia Statistics Agency, Ministry of Health Zambia, ICF. Zambia Demographic and Health Survey 2018. In. Lusaka, Zambia, and Rockville, Maryland, USA: Zambia Statistics Agency, Ministry of Health, and ICF; 2019.
3. Brown T, Peerapatanapokin W. Evolving HIV epidemics: the urgent need to refocus on populations with risk. *Curr Opin HIV AIDS.* 2019;14(5):337-353.
4. Kharsany AB, Karim QA. HIV Infection and AIDS in Sub-Saharan Africa: Current Status, Challenges and Opportunities. *Open AIDS J.* 2016;10:34-48.
5. Haberer JE, Mugo N, Baeten JM, Pyra M, Bukusi E, Bekker LG. PrEP as a Lifestyle and Investment for Adolescent Girls and Young Women in Sub-Saharan Africa. *J Int Assoc Provid AIDS Care.* 2019;18:2325958219831011.
6. Mugo NR, Ngure K, Kiragu M, Irungu E, Kilonzo N. The preexposure prophylaxis revolution; from clinical trials to programmatic implementation. *Curr Opin HIV AIDS.* 2016;11(1):80-86.
7. Baeten JM, Donnell D, Ndase P, et al. Antiretroviral prophylaxis for HIV prevention in heterosexual men and women. *The New England journal of medicine.* 2012;367(5):399-410.
8. Grant RM, Lama JR, Anderson PL, et al. Preexposure chemoprophylaxis for HIV prevention in men who have sex with men. *The New England journal of medicine.* 2010;363(27):2587-2599.
9. Thigpen MC, Kebaabetswe PM, Paxton LA, et al. Antiretroviral preexposure prophylaxis for heterosexual HIV transmission in Botswana. *The New England journal of medicine.* 2012;367(5):423-434.
10. Van Damme L, Corneli A, Ahmed K, et al. Preexposure prophylaxis for HIV infection among African women. *The New England journal of medicine.* 2012;367(5):411-422.
11. Marrazzo JM, Ramjee G, Richardson BA, et al. Tenofovir-Based Preexposure Prophylaxis for HIV Infection among African Women. *New England Journal of Medicine.* 2015;372(6):509-518.
12. Fonner VA, Dalglish SL, Kennedy CE, et al. Effectiveness and safety of oral HIV preexposure prophylaxis for all populations. *AIDS (London, England).* 2016;30(12):1973-1983.
13. Grant RM, Lama JR, Anderson PL, et al. Preexposure chemoprophylaxis for HIV prevention in men who have sex with men. *The New England journal of medicine.* 2010;363(27):2587-2599.
14. Dimitrov DT, Mâsse BR, Donnell D. PrEP Adherence Patterns Strongly Affect Individual HIV Risk and Observed Efficacy in Randomized Clinical Trials. *J Acquir Immune Defic Syndr.* 2016;72(4):444-451.
15. WHO. Guideline on when to start antiretroviral therapy and on pre-exposure prophylaxis for HIV. 2015. https://www.who.int/hiv/pub/guidelines/earlyrelease-arv/en/.
16. Bailey JL, Molino ST, Vega AD, Badowski M. A Review of HIV Pre-Exposure Prophylaxis: The Female Perspective. *Infectious diseases and therapy.* 2017;6(3):363-382.
17. Valente PK, Mantell JE, Masvawure TB, et al. "I Couldn't Afford to Resist": Condom Negotiations Between Male Sex Workers and Male Clients in Mombasa, Kenya. *AIDS and behavior.* 2019.
18. Haberer JE, Bangsberg DR, Baeten JM, et al. Defining success with HIV pre-exposure prophylaxis: a prevention-effective adherence paradigm. *AIDS (London, England).* 2015;29(11):1277-1285.
19. Hendrickson C, Long L, van de Vijver D, et al. Novel metric for evaluating pre-exposure prophylaxis programme effectiveness in real-world settings. *Lancet HIV.* 2020;7(4):e294-e300.
20. National HIV/AIDS/STI/TB Council Zambia. National AIDS Strategic Framework (NASF) 2017-2021. 2017. https://www.nac.org.zm/sites/default/files/publications/NASF%202017%20-%202021.pdf.
21. South African National Department of Health. *Oral PrEP Monitoring and Evaluation (M&E) Guidelines.* Pretoria: South African National Department of Health;2017.
22. WHO. WHO expands recommendation on oral pre-exposure prophylaxis of HIV infection (PrEP). 2015. https://www.who.int/hiv/pub/prep/policy-brief-prep-2015/en/.

23. Ministry of Health Zambia. Zambia Consolidated Guidelines for Prevention and Treatment of HIV Infection. In. Lusaka, Zambia: Ministry of Health; 2016.
24. Ministry of Health Zambia. Zambia Consolidated Guidelines for Prevention and Treatment of HIV Infection. In. Lusaka, Zambia: Ministry of Health; 2018.
25. Ministry of Health Zambia. Implementation Framework & Guidance for Pre-Exposure Prophylaxis of HIV Infection. In. Lusaka, Zambia: Ministry of Health; 2018.
26. CDC. Understanding the HIV Care Continuum. 2019. https://www.cdc.gov/hiv/pdf/library/factsheets/cdc-hiv-care-continuum.pdf.
27. WHO. Implementation Tool for Pre-Exposure Prophylaxis (PrEP) of HIV Infection. 2018. https://www.who.int/hiv/pub/prep/prep-implementation-tool/en/.
28. Dunbar MS, Kripke K, Haberer J, et al. Understanding and measuring uptake and coverage of oral pre-exposure prophylaxis delivery among adolescent girls and young women in sub-Saharan Africa. *Sex Health.* 2018;15(6):513-521.
29. Nunn AS, Brinkley-Rubinstein L, Oldenburg CE, et al. Defining the HIV pre-exposure prophylaxis care continuum. *AIDS (London, England).* 2017;31(5):731-734.
30. Liu A CG, Cohen S, Bacon O, Kolber M, Amico K, Mugaveri M, Grant R, Buchbinder S. The Spectrum of Engagement in HIV Prevention: Proposal for a PrEP cascade. 7th International Conference on HIV Treatment and Prevention Adherence; 2012; Miami, FL.
31. Zambia P. Country Operational Plan 2020 Strategic Direction Summary. In:2020.
32. Coleman R. Setting the scene, setting the targets. The Joint United Nations Programme on HIV/AIDS prevention targets of 2016 and estimating global pre-exposure prophylaxis targets. *Sex Health.* 2018;15(6):485-488.
33. WHO C, UNAIDS, FHI360. Biobehavioral survey guidelines for Populations at Risk for HIV. 2017. https://apps.who.int/iris/bitstream/handle/10665/258924/9789241513012-eng.pdf?sequence=1.
34. Smith PJ. Bayesian methods for multiple capture-recapture surveys. *Biometrics.* 1988;44(4):1177-1189.
35. Smith P. Bayesian analyses for a multiple capture-recapture model. *Biometrika.* 1991;78(2):399-407.
36. Yu D, Calleja JM, Zhao J, Reddy A, Seguy N. Estimating the size of key populations at higher risk of HIV infection: a summary of experiences and lessons presented during a technical meeting on size estimation among key populations in Asian countries. *Western Pac Surveill Response J.* 2014;5(3):43-49.
37. Dunkle KL, Stephenson R, Karita E, et al. New heterosexually transmitted HIV infections in married or cohabiting couples in urban Zambia and Rwanda: an analysis of survey and clinical data. *Lancet.* 2008;371(9631):2183-2191.
38. Irungu EM, Baeten JM. PrEP rollout in Africa: status and opportunity. *Nat Med.* 2020;26(5):655-664.
39. UNAIDS. UNAIDS Country Facsheet Zambia. 2019. https://www.unaids.org/en/regionscountries/countries/zambia.
40. AVERT. HIV and AIDS in Zambia. 2018. https://www.avert.org/printpdf/node/408.
41. Nichols BE, Boucher CA, van Dijk JH, et al. Cost-effectiveness of pre-exposure prophylaxis (PrEP) in preventing HIV-1 infections in rural Zambia: a modeling study. *PLoS One.* 2013;8(3):e59549.
42. Verguet S, Stalcup M, Walsh JA. Where to deploy pre-exposure prophylaxis (PrEP) in sub-Saharan Africa? *Sex Transm Infect.* 2013;89(8):628-634.
43. Kim AA, Behel S, Northbrook S, Parekh BS. Tracking with recency assays to control the epidemic: real-time HIV surveillance and public health response. *AIDS (London, England).* 2019;33(9):1527-1529.
44. Gumede-Moyo S, Todd J, Bond V, Mee P, Filteau S. A qualitative inquiry into implementing an electronic health record system (SmartCare) for prevention of mother-to-child transmission data in Zambia: a retrospective study. *BMJ Open.* 2019;9(9):e030428.
45. DHIS-2. Individual Data Records with DHIS-2 Tracker. 2019. https://www.dhis2.org/tracker.
46. PEPFAR. Monitoring, Evaluation and Reporting Indicator Reference Guide. MER 2.0 (Version 2.4). September 2019. https://datim.zendesk.com/hc/en-us/articles/360000084446-MER-Indicator-Reference-Guides.

47. Grant RM, Anderson PL, McMahan V, et al. Uptake of pre-exposure prophylaxis, sexual practices, and HIV incidence in men and transgender women who have sex with men: a cohort study. *Lancet Infect Dis.* 2014;14(9):820-829.
48. Donnell D, Baeten JM, Bumpus NN, et al. HIV protective efficacy and correlates of tenofovir blood concentrations in a clinical trial of PrEP for HIV prevention. *J Acquir Immune Defic Syndr.* 2014;66(3):340-348.
49. Gilead Sciences. Truvada Package Insert. 2020. https://www.gilead.com/-/media/files/pdfs/medicines/hiv/truvada/truvada_pi.pdf.
50. ZAMRA. Guidelines for detecting and reporting adverse drug or vaccine reactions and events in Zambia. 2006. http://www.zamra.co.zm/wp-content/uploads/2016/10/Guidelines-on-Pharmacovigilance.pdf.

The printing of this document was made possible by the support of the American people through President's Emergency Plan for AIDS Relief ***(PEPFAR)*** under the terms of Centers for Disease Control and Prevention ***(CDC)*** Cooperative Agreement No ***NU2GGH002123-03-00.***

Community Impact to Reach Key and Underserved Individuals for Treatment and Support ***(CIRKUITS)*** managed by University of Maryland ***(UMB)***.

The contents of this document are the responsibility of the Ministry of Health, and not necessarily reflect the views of CDC or the United States Government
